# Harnessing historical genebank data to accelerate pea breeding

**DOI:** 10.1007/s00122-025-05032-5

**Published:** 2025-09-09

**Authors:** Lanique Niels, Jochen Christoph Reif, Lars-Gernot Otto, Vilson Mirdita, Markus Oppermann, Ulrike Lohwasser, Matthias Kotter, Stephan Weise, Samira El Hanafi

**Affiliations:** Leibniz Institute of Plant Genetics and Crop Plant Research (IPK), Gatersleben, Germany

## Abstract

The German Federal Ex Situ Genebank for Agricultural and Horticultural Crops (IPK) harbours over 3000 pea plant genetic resources (PGRs), backed up by corresponding information across 16 key agronomic and economical traits. The unbalanced structure and inconsistent format of this historical data has precluded effective leverage of genebank accessions, despite the opportunities contained in its genetic diversity. Therefore, a three-step statistical approach founded in linear mixed models was implemented to enable a rigorous and targeted data curation. Spring accessions revealed considerable breeding potential, with protein content exceeding market standards by almost one-fifth and with hundred grain weight that could match the upper limits reported for European elite varieties. This variation is embedded within structured populations, comprising five convarieties including sugar snaps and field pea, adding value for breeding across diverse morphotypes and market segments. Winter accessions demonstrated cold resilience, with post-winter survival rate up to 79.27% under minimum temperatures as low as − 17.1 °C. This variation is of particular relevance given the limited availability of winter-hardy cultivars able to evade summer drought and heat stresses. Transformation of the IPK Genebank into a bio-digital resource redirects formerly static material into central leverage for plant breeding in view of contemporary challenges. As such, this investigation activated the IPK pea population for use in among others breeding for a wide variety of ideotypes, research into adaptation, and future combination with omics studies.

**Supplementary Information:**

The online version contains supplementary material available at 10.1007/s00122-025-05032-5.

## Introduction

Despite being the backbone of global crop diversity conservation, most plant genetic resources (PGRs) remain poorly characterized and largely untapped in modern breeding. This disconnect persists even though searches of collections for traits of interest have identified suitable donor material in various cases, resulting in introgressions that addressed pressing issues (Guerra et al. 2022; Roorkiwal et al. 2020; Schulthess et al. 2022). Whereas genebank genetic diversity is plentiful and continues to offer the raw material needed to adapt crops to climate stress, market demands, and sustainability goals, the challenge lies in access, visibility and usability (Dempewolf et al. 2017; He et al. 2022; McCouch et al. 2013). Therefore, pre-breeders and breeders have often turned to more narrowly defined breeding collections, comprising selected landraces and a few promising accessions from public collections (Engels et al. 2024). This reliance has intensified over time, particularly following the implementation of the Nagoya protocol, which, despite its role in promoting fairness, has introduced complexities in accessing and exchanging genetic resources. As a result, breeding programmes increasingly prioritize internally managed collections, while vast pools of diversity conserved in public genebanks, especially in underutilized crops, remain underexplored (Engels et al. 2024).

Pea (*Pisum sativum*), a high yielding temperate climate legume with valuable grain traits (Annicchiarico 2008; Carrouée et al. 2003; Crosta et al. 2023; Rogers et al. 2024; Thavarajah et al. 2023; Windsor et al. 2024), exemplifies this paradox. Despite its advantageous traits, it remains a minor crop due to marginal profitability compared to cereals (Magrini et al. 2016; Rubiales et al. 2021a) and soybean (FAO 2024; Rogers et al. 2024). Particularly, winter pea holds potential to increase relevance of pea cultivation due to its early sowing, season extension, and nitrogen savings, but is constrained by its sensitivity to frost and insufficient winter hardiness. In a recent study, seedlings of elite genotypes demonstrated an expected 50% survival between − 11.6 and − 14.5 °C (Franguelli et al. 2024), exceeding commonly reported tolerated ranges between − 8 and − 13 °C (Liu et al. 2017; Urbatzka et al. 2012; Vogt-Kaute and Quendt 2022). Continuation and expansion of winterhardiness is essential to enable reliable stand establishment and yield stability in cold-prone environment. Moreover, for spring and winter pea alike, development of more profitable varieties with the focus on yield, quality traits and resilience is essential, but is hampered by limited genetic diversity in the elite genepools, resulting from decades of selection (Annicchiarico et al. 2017). The untapped genetic wealth of PGRs readily available in genebanks offers a potential solution (Qiu et al. 2013), provided we can overcome as a first step the bottlenecks in data usability. While many accessions have been phenotyped during regeneration periods, their associated data are often unbalanced, inconsistent, and unsuitable for direct use in breeding decisions (Anglin et al. 2018).

Therefore, curation of genebank data to meet the FAIR principles (Findable, Accessible, Interoperable, and Reusable; Wilkinson et al. 2016) is critical to transforming this underutilized wealth into an engine of crop innovation (Anglin et al. 2018). Historical data, once curated, can mimic long-term, multi-environment trials and explore trait plasticity, environmental adaptation, and rare variation. This potential has been demonstrated through initiatives at the IPK Genebank (González et al. 2018; Philipp et al. 2018), where curated records have enabled genomic studies to unlock new sources of variation and guide introgression strategies (Berkner et al. 2024b; El Hanafi et al. 2023; Jiang et al. 2021; Milner et al. 2019). Considering the broad variety of environmental and consumer demands, continuation and expansion of this trend is essential. Therefore, the search for new sources of diversity as input for research and breeding is as relevant as ever.

In line with this overarching need, the IPK Genebank’s pea collection presents a valuable case study. As the sixth-largest and most diverse pea repository globally (Bagheri et al. 2023), it comprises over 5300 accessions including 109 winter pea accessions. Of these, respectively 3041 spring and 45 winter accessions are associated to detailed phenotype data, gathered from 76 and 7 countries. To overcome the limits imposed by its unbalanced historical data, advanced statistical methods are required to correct biases and extract meaningful patterns. Unlocking this dataset’s full potential will support the development of climate-resilient and high-performing varieties, strengthening pea’s role in sustainable agriculture. Therefore, the following objectives were to: (1) unlock the historical pea data of the IPK Genebank by utilizing a three-step data curation strategy; (2) characterize phenotypic variation across spring and winter accessions; (3) identify frost-tolerant winter accessions for future breeding use; and (4) evaluate convarietal differentiation and trait interdependence to inform pre-breeding strategies.

## Materials and methods

### Plant material

This study focuses on 3086 pea accessions maintained at the German Federal Ex Situ Genebank at the Leibniz Institute of Plant Genetics and Crop Plant Research (IPK), Germany (Weise et al. 2025). Acquired through expeditions, seed exchanges, and breeding programmes, the collection spans a broad geographical range (Online Resource 1). Of these accessions, 2988 were classified as *Pisum sativum sativum* and include various convarieties according to Alefeld’s classification: 1043 *sativum* (garden pea), 590 *speciosum* (field pea), 163 *axiphium* (snow pea), 900 *medullare* (wrinkled pea with high amylose content), and 30 *medullosaccharatum* (an intermediate form between medullare and axiphium; Alefeld 1866; Green 2008; Lehmann 1954; Osman and Ali 2021). In total 45 accessions were identified as winter type, characterized by a delayed growth when spring-sown and reliable winter survival after autumn sowing. From these, 38 belong to *P. sativum sativum speciosum* convariety. The passport data for these accessions are documented in the IPK Genebank Information System (GBIS; Oppermann et al. 2015).

### Field trials

The phenotypic data were routinely collected at Gatersleben, Germany (latitude: 51° 49′ 22.5′′ N, longitude: 11° 16′ 40.6′′ E, 110.5 m.a.s.l.) during the seed regeneration process, which was carried out when seed stocks became insufficient or seed viability fell below a critical threshold. Regeneration trials followed an experimental design with no replication randomized across regeneration years, with a plot size of 2.88 m^2^. The standard sowing rate was 100 seeds per plot, although limited seed availability occasionally required a reduced sowing rate. Additionally, in 2009, 2021, 2022, and 2023, a total of 9, 19, 29, and 22 spring accessions, respectively, were grown in Greenhouse facilities. The spring peas were sown between March and May, with data spanning from 1946 to 2023 (Online Resource 2). On average, each spring pea accession was evaluated in 4.36 years. Winter peas were sown in september or october, with most observations (84.72%) collected between 1955 and 1961. On average, each winter pea accession was tested in 4.8 years.

Phenotypic data were collected for 16 quantitative traits including phenological, agronomic, and yield-related traits (Table 1). Hundred grain weight (HGW) and protein content (Prot) of spring accessions were measured separately in a distinct trial setup in 1976. Accessions showing favourable performance for these two traits were regrown in 1978, and a subset was further evaluated in 1980 to assess performance across years (Müntz and Lehmann 1987).

**Table 1:** List of the 16 quantitative traits phenotyped by the IPK Gatersleben for the 3041 spring and 45 winter pea accessions, respectively.

Trait	Unit	Definition
Days to emergence (EmDays)	Days	Days at which 50% of the plants at the plot emerged, after March 1st for spring (*S*) or after sowing (*W*)
Start of flowering time (FTS)		Days after March 1st (*S*) or January 1st (*W*) at which the first flower opened for 50% of the plants in the plot
End of flowering time (FTE)		Days after March 1st (*S*) or January 1st (*W*) at which the last flower opened for 50% of the plants in the plot
Date of picking ripeness (Ripe)		Days after March 1st (*S*) or January 1st (*W*) at which 50% of the plants in the plot the first pod was suitable for commercial harvest
Duration of flowering (FTdur)		Number of days between the start and the end of flowering (*S* and *W*)
Plant height at flowering time (PHFT)	cm	Straight distance between the tip of the shoot and the soil, measured when 50% of the plants flowered and averaged across the plot (*S* and *W*)
Plant height (minimum) (PHmin)		Straight distance between the tip of the shoot and the soil of the shortest plant measured when 50% of the plot flowered, recorded in plots with accessions with variable heights (*S* and *W*)
Plant height (maximum) (PHmax)		Straight distance between the tip of the shoot and the soil of the tallest plant measured when 50% of the plot flowered, recorded in plots with accessions with variable heights (*S* and *W*)
Height of the first pod (PHpod)	cm	Height at the point where the stalk carrying the first pod branches off from the stem, averaged across different plants (*S* and *W*)
Number of flowers per inflorescence (Flr)	–	Classes indicating the number of flowers per inflorescence (1 = one, 2 = one or two, 3 = two, 4 = two or three, 5 = three or more), averaged across the plot (*S*)
Seed size (Ss)	–	Classes indicating the size of the seeds harvested from the entire plot (1 = small, 2 = medium, 3 = large; *S*)
Number of seeds per pod (Spp)	–	Average number of seeds per pod in 8 pods of 8 different plants (*S*)
Hundred grain weight (HGW)	g	Weight of one hundred grains, obtained by averaging the weight of 3 times 100 pea grains (*S* and *W*)
Protein content (Prot)	%	Protein content measured with the Kjeldahl method in air-dried pea grains (*S*)
Survival rate (Surv)		Number of vital plants observed in a row base within a plot, measured before and after winter (post-winter survival rate, PWSurv). Scored on 1–9 scale, and converted into percentage (*W*)

(*S*) and (*W*) indicate traits measured during spring and winter cultivation

Detailed environmental data were retrieved from a local weather station, including daily records of temperature, precipitation, and snow cover for the periods between November 1th to April 30th. Implausible minimum temperature values deviating by more than 20 °C from either adjacent days or by more than 15 °C from the corresponding minimum temperature at 2 m above ground were removed. Missing values were then imputed using the na_kalman function from the R package imputeTS (version 3.3; Moritz and Bartz-Beielstein 2017) or using a model adapted from Trnka et al. (2010) in case of snow cover with known precipitation.

### Data analyses

Phenotypic data of spring and winter accessions was analysed separately. Plausibility checks were performed initially to exclude clearly erroneous observations. Observations falling outside of the range of the mean three times the standard deviation were excluded. Traits such as days to emergence (EmDays), start and end of flowering (FTS and FTE), and picking ripeness (Ripe) that exhibited unusually early or late values were flagged and discarded if they either (i) indicated an abnormal growing season, or (ii) were inconsistent with the patterns observed for the other three traits.

The following linear mixed model (LMM) was fitted to analyse the historical phenotypic data of each trait using the ASReml package in R (version 4.2.0.267; Butler et al. 2017; R Core Team 2024):

1 $$y_{{{\text{ijkl}}}} = \, \mu \, + \, g_{{\text{i}}} + \, a_{{\text{j}}} + \, t_{{\text{k}}} + \, v_{{\text{l}}} + \, g_{{\text{i}}} :t_{{\text{k}}} + \, e_{{{\text{ijkl}}}}$$

where *y*_ijkl_ is the phenotypic records for the ith accession tested in the jth year, within the kth experiment of the lth convariety. *μ* denotes the intercept, *g*_i_ the effect of the ith accession, *a*_j_ the effect of the jth year, *t*_k_ the effect of the kth experiment, and *v*_l_ the effect of the lth convariety. The term *g*_i_:*t*_k_ captures the interaction between the genotype and experiment, and *e*_ijkl_ represents the residual. Full and reduced models were applied depending on the trait and the model convergence. *v*_l_ was omitted if its inclusion did not lead to significant improvement of the model fit.

Filtered data after plausibility check underwent a two-step outlier correction procedure. The first step involved year-based outlier detection to identify years with highly deviating values. Model 1 was fitted assuming accessions as random and years as fixed effects. Year effects and year-specific error effects were used to calculate the coefficient of variation (CV). If the CV for a given year exceeded three standard deviations from the mean CV, the corresponding trait-year records were removed. The second step involved record-based outlier correction, where model 1 was applied to the pre-corrected dataset from step one, with a slightly different assumption considering accessions as fixed and years as random effects. Standardized residuals were assessed, and any data points that fell beyond the Tukey-defined threshold were excluded (Bernal-Vasquez et al. 2016; Philipp et al. 2018).

Subsequently, Eq. (1) was re-fitted again to compute variance components and broad-sense heritability, as well as to calculate the best linear unbiased predictions (BLUEs). For BLUEs computation, the same assumptions in Eq. (1) as specified for outlier correction were considered, although convariety effects were omitted to retain differences in performance among accessions. Broad-sense heritability was computed assuming all factors as random effects except the intercept, with year and experiment combined into a single factor. To account for the unbalanced data, ad-hoc heritability was calculated as follows (Holland et al. 2003):

2 $$h^{2} = \frac{{\sigma_{g}^{2} }}{{\sigma_{g}^{2} + \frac{{\sigma_{gb}^{2} }}{q} + \frac{{\sigma_{e}^{2} }}{p}}} ,$$

where $q = \mathop \sum \limits_{i = 1}^{n} \frac{{g_{{\text{i }}} :b_{{\text{j}}} }}{i}$ is the sum of each year *x* genotype interaction effect divided by their index, and $p = \mathop \sum \limits_{i = 1}^{n} \frac{{e_{{i{\text{jl}}}} }}{i}$ is the sum of each residual effect divided by their index. In the case of a reduced model, $\frac{{\sigma_{gb}^{2} }}{q}$ was omitted.

Convariety effects on BLUEs were calculated by fitting:

3 $$y_{{{\text{ijl}}}} = \, \mu \, + \, v_{{\text{l}}} + \, e_{{{\text{ijl}}}}$$

Based on this model, the R package emmeans (version 1.10.4; Lenth 2024) was used to determine the significance level of pairwise differences with Holm’s correction for multiple testing.

To assess the relationships between traits, Pearson’s correlation coefficients for the resulting BLUEs of each trait pair within both winter and spring collections were further assessed using the R package WGCNA (1.72–5, Langfelder and Horvath 2008, 2012). Significance levels were adjusted with Holm’s correction for multiple testing.

## Results

### Data curation improved the quality of the historical pea data

A three-step outlier correction strategy was implemented to enhance the quality of the IPK historical phenotypic data. As a result, an average of 1.3 and 0.2% of the accessions were excluded from the spring and winter populations across traits, respectively (Tables 2 and 3, Online Resources 3 and 4). Despite the relatively small proportion of the removed data, Heritability estimates increased substantially for some traits, particularly in the spring pea population, where estimates rose by up to 903% (Tables 2 and 3). This suggests that a small fraction of inconsistent data points had disproportionately inflated residual variance, obscuring meaningful differences in accession performance. In general, higher heritabilities were observed in the spring compared to the winter population, likely due to the larger number of spring accessions and trial years, which reduced environmental variability and improved performance estimates. Across both populations, phenology‐related traits tended to show stronger genetic control, whereas certain traits in the winter population, such as Ripe and EmDays, exhibited very low heritability, highlighting the significant influence of environmental factors under winter conditions (Online Resource 5). Meanwhile, agronomic traits consistently displayed moderate-to-high heritability in both populations, indicating a consistent genetic contribution despite varying environmental effects.

**Table 2:** The estimated broad-sense heritability before and after the exclusion of outliers for each trait in the spring population

	*N*_data_	*N*_outlier_	*N*_geno_	*N*_geno͡ out_	Initial $h^{2}$	Final $h^{2}$	$\Delta h^{2}$(%)
Days to emergence	11,653	280 (2.35%)	2941	49 (1.64%)	0.07	0.75	902.63
Start of flowering	11,436	277 (2.36%)	2936	54 (1.81%)	0.83	0.91	9.92
End of flowering	10,788	237 (2.15%)	2847	36 (1.25%)	0.68	0.79	16.64
Date of picking ripeness	9545	178 (1.83%)	2580	7 (0.27%)	0.68	0.81	18.55
Duration of flowering	10,704	186 (1.71%)	2863	10 (0.35%)	0.33	0.47	42.66
Plant height at flowering time	10,449	198 (1.86%)	2919	15 (0.51%)	0.87	0.90	3.3
Plant height minimal	687	46 (6.28%)	425	1 (0.23%)	0.83	0.92	10.42
Plant height maximal	797	63 (7.33%)	544	4 (0.73%)	0.84	0.93	11.54
Height of the first pod	8746	188 (2.1%)	2726	11 (0.4%)	0.78	0.83	6.41
Number of flowers per inflorescence	10,478	137 (1.29%)	2982	1 (0.03%)	0.69	0.74	7.52
Seed size	10,609	201 (1.86%)	2762	1 (0.04%)	0.72	0.77	7.1
Number of seeds per pod	8906	148 (1.63%)	2634	6 (0.23%)	0.52	0.60	14.3
Protein content	1719	167 (8.85%)	1283	70 (5.17%)	0.44	0.69	57.21
Hundred grain weight	1706	176 (9.35%)	1293	60 (4.43%)	0.85	0.92	8.95

**Table 3:** The estimated broad-sense heritability before and after the exclusion of outliers for each trait in the winter population

	*N*_data_	*N*_outlier_	*N*_geno_	*N*_geno͡ out_	Initial $h^{2}$	Final $h^{2}$	$\Delta h^{2}$(%)
Days to emergence	313	3 (0.95%)	45	0 (0%)	0.00	0.00	0.98
Start of flowering	161	6 (3.59%)	42	0 (0%)	0.61	0.85	39.6
End of flowering	100	3 (2.91%)	38	0 (0%)	0.41	0.32	− 23.15
Date of picking ripeness	84	5 (5.62%)	38	0 (0%)	0.00	0.00	NA
Duration of flowering	99	3 (2.94%)	38	0 (0%)	0.00	0.21	NA
Plant height at flowering time	106	2 (1.85%)	35	0 (0%)	0.72	0.76	5.52
Plant height minimal	106	2 (1.85%)	35	0 (0%)	0.70	0.79	12.5
Plant height maximal	104	2 (1.89%)	35	0 (0%)	0.70	0.77	10.23
Height of the first pod	17	2 (10.53%)	11	0 (0%)	0.87	0.94	7.59
Hundred grain weight	116	2 (1.69%)	38	0 (0%)	0.75	0.85	13.16
Survival rate	159	5 (3.05%)	38	1 (2.56%)	0.00	0.11	NA
Post-winter survival rate	158	2 (1.25%)	39	0 (0%)	0.64	0.84	30.34

NA values indicate cases where no genotypic variance was detected, resulting in zero heritability

### Winter pea accessions exhibited substantial winterhardiness

**Fig. 1:**
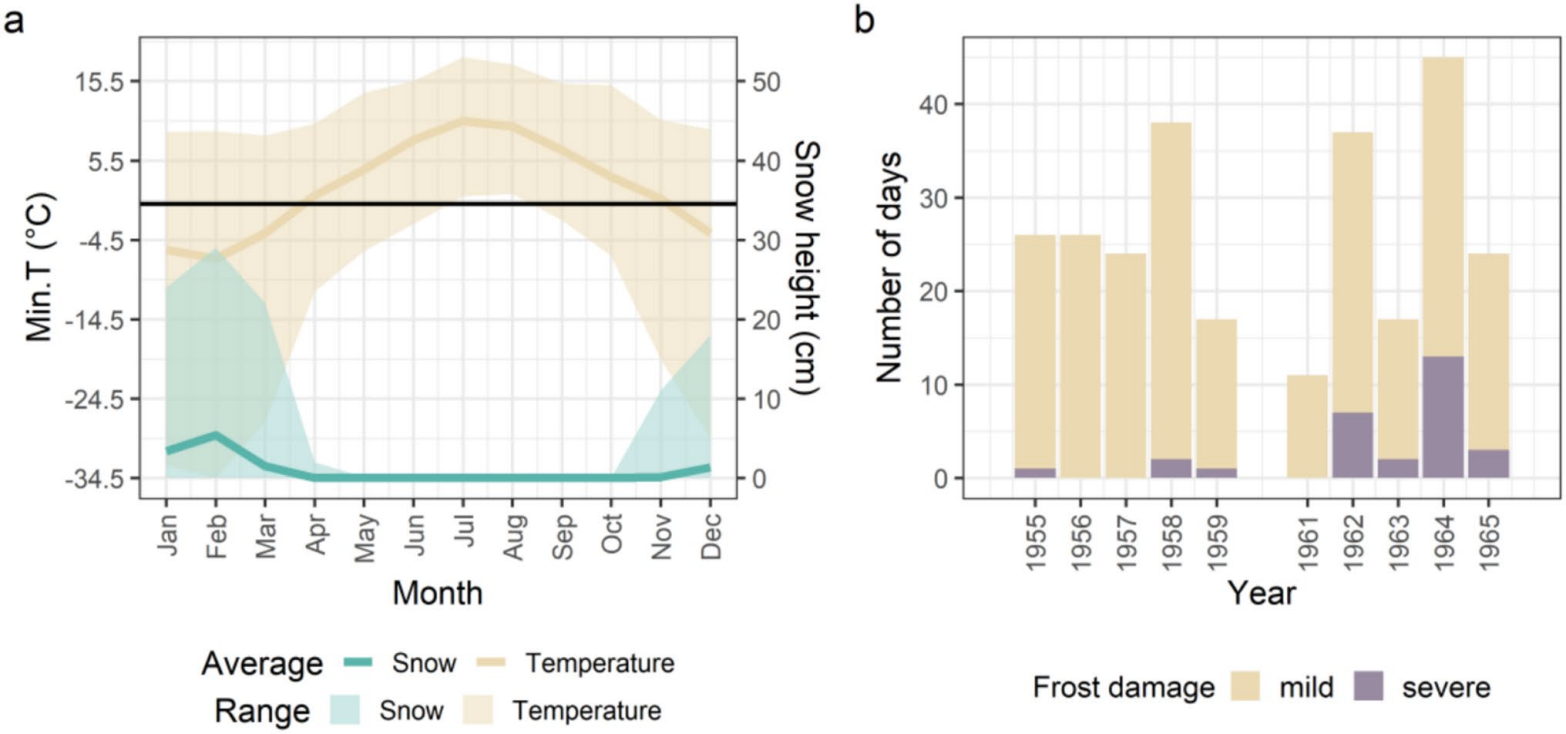
Overview of the weather data during years with winter pea cultivation. **a** Monthly averages and ranges of the lowest daily temperatures (yellow) and snow cover (blue) measured 5 cm above ground from 1955 to 1965 (excluding 1960). **b** Number of days per year with mild (minimum daily temperature at 5 cm above ground < − 6 °C) and severe (minimum daily temperature at 5 cm above ground < − 14 °C) frost stress under snow cover less than 5 cm

**Fig. 2:**
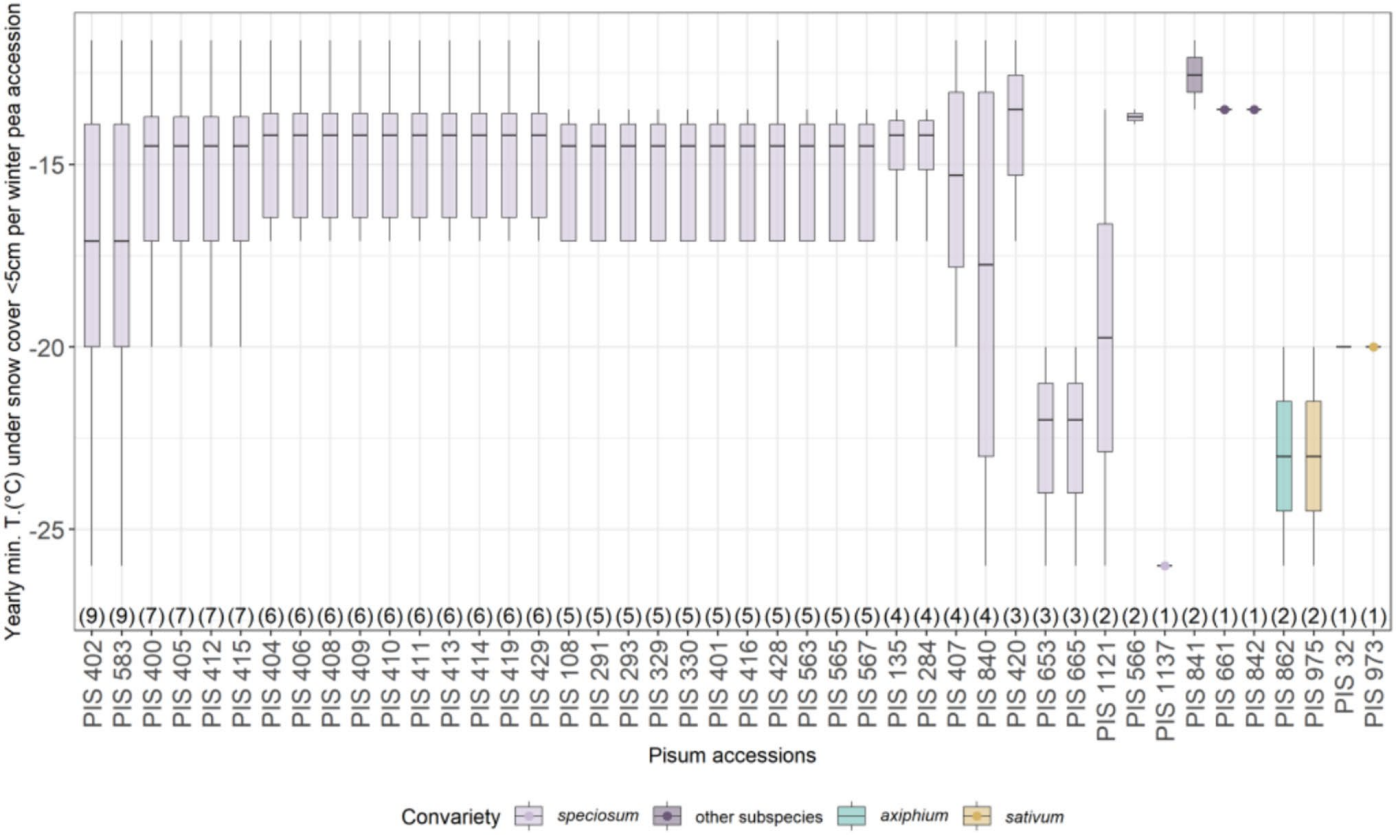
Yearly minimum temperature at 5 cm above ground under snow cover < 5 cm for each winter pea accession. Numbers between brackets indicate the number of cultivation years per accession

**Fig. 3:**
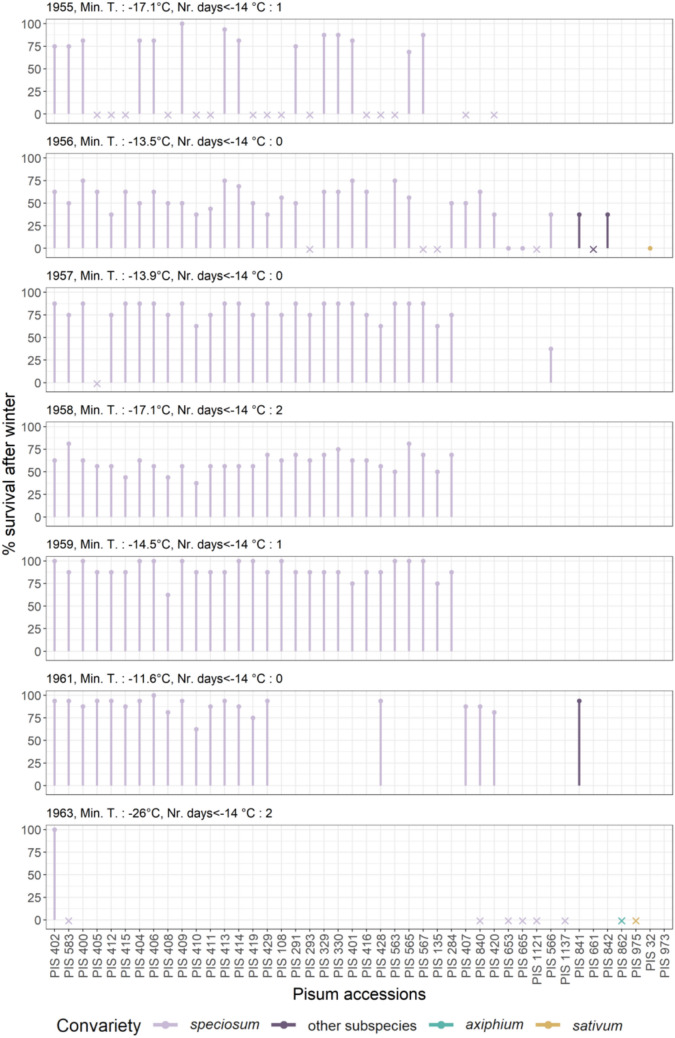
Percentage post-winter survival (PWSurv) of each winter pea accession across 8 years. Crosses indicate years in which an accession was phenotyped for at least one post-winter trait, but PWSurv was not recorded. The yearly lowest minimum temperature at 5 cm above ground and number of days at which this temperature reached below − 14 °C are shown above each graph

From December to March, the minimum temperature measured at 5 cm above ground at Gatersleben location consistently remained below 0 °C across years, with February being on average the coldest month (Fig. 1a). Severe frost events, defined as temperatures below − 14 °C with less than 5 cm snow cover, occurred in 7 out of the 10-year periods (Fig. 1b) in the timeframe between November 1st of the sowing year to April 30th in the following year. Due to the non-orthogonal design of the trials, winter pea accessions were exposed to varying levels of cold stress (Fig. 2, Online Resource 6). For example, accession PIS 402 experienced cold stress over 9 years, offering robust evidence of its consistent winterhardiness under recurrent extreme conditions. Notably, several accessions of the *axiphium* convariety (e.g. PIS 862) and *sativum* convariety (e.g. PIS 975 and PIS 32) survived at minimum temperature measured at 5 cm height below − 20 °C with less than 5 cm snow cover (Fig. 3, Online Resource 7), highlighting their potential as valuable genetic resources for enhancing frost tolerance. Additionally, the *speciosum* convariety consistently demonstrated superior survival compared to others. These results surpass earlier thresholds reported from large-scale trials (− 10 to − 18 °C; Liu et al. 2017; Zhang et al. 2016), from field trials with snow cover (− 20.3 °C; Homer et al. 2016) and from controlled settings (− 8 to − 14.5 °C; Franguelli et al. 2024; Homer et al. 2016), underscoring the exceptional frost tolerance documented in this study. The enhanced cold tolerance was independent of country of provenance, given that the site of collection or of evolutionary origin might differ. As such, survival rates were similarly high among accessions collected from diverse latitudes (e.g. Greece, Albania, and Germany).

### Broad phenotypic variation to unlock trait-specific breeding improvement

**Fig. 4:**
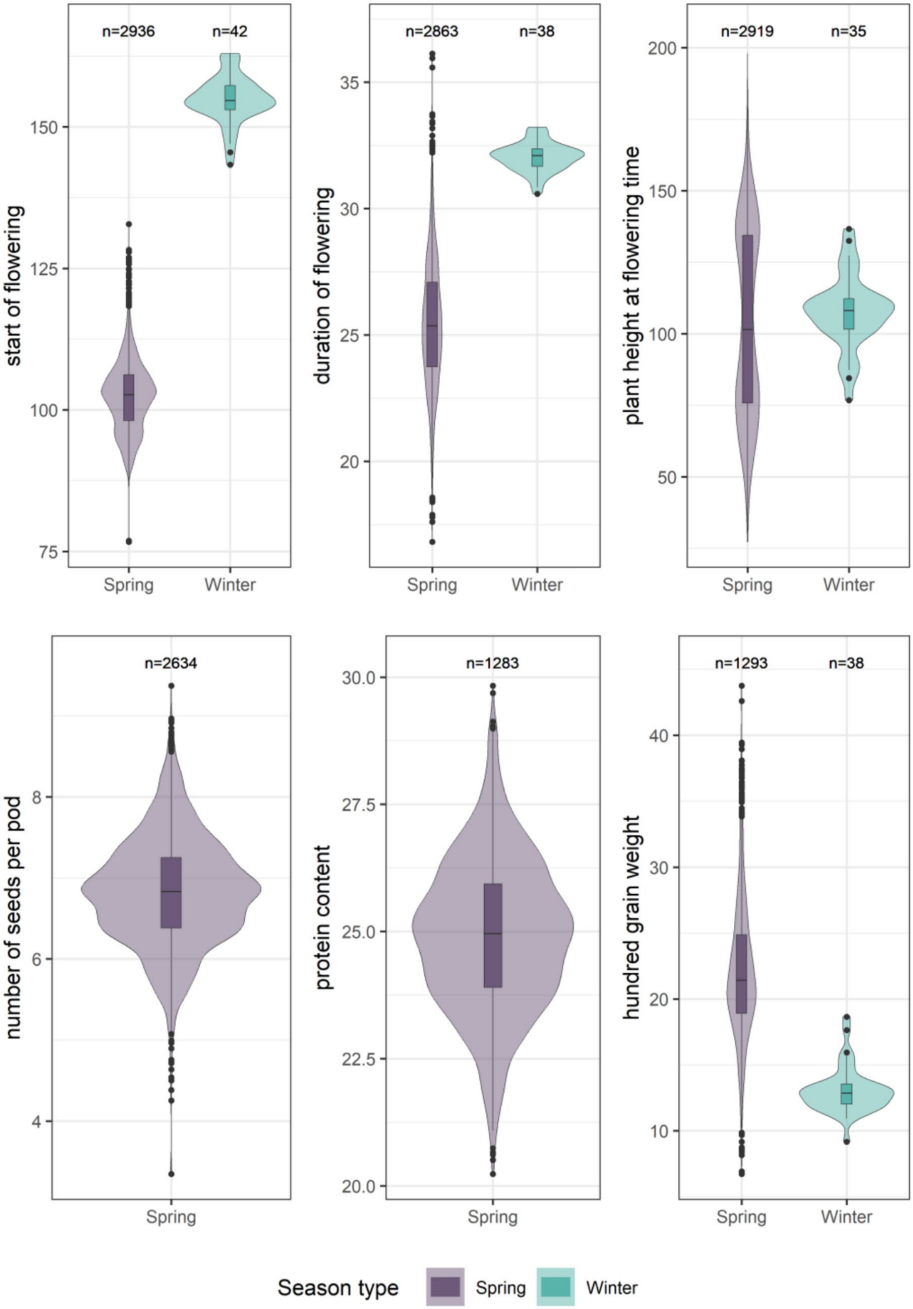
Distribution of BLUEs for the traits start of flowering (days), duration of flowering (days), plant height (cm), number of seeds per pod, protein content (%), and hundred grain weight (g) for spring and winter accessions. The number of BLUEs is indicated above the corresponding graphs

To investigate the variation present within the IPK Genebank, BLUEs were calculated for each accession. The traits related to earliness, height, yield components, and post-winter survival rate (PWSurv) were prioritized for further analyses. Information of the remaining traits is presented in online resource 8. Distinct patterns emerged between spring and winter accessions, reflecting their phenotypic diversity and adaptability (Fig. 4). Spring accessions consistently exhibited a broader range of phenotypic variation across traits. For instance, HGW in spring accessions ranged from 6.72 to 43.75 g, representing a fourfold increase compared to the range observed in winter accessions, underscoring its potential for yield improvement. Moreover, spring accessions reached a Prot up to 29.83%, making them ideal for quality-focused breeding efforts.

Winter accessions, on the other hand, exhibited strong winterhardiness and capitalized on a longer flowering window, highlighting their adaptability to adverse conditions. Notably, PWSurv showed substantial variability, ranging from 30.34 to 79.27%, emphasizing the importance to prioritize selection for winterhardiness in pea breeding programmes (online resource 8). The limited phenotypic variation observed in winter populations, except for PWSurv, likely reflects both their smaller population size and the selective constraints imposed by winter conditions. Overall, these findings underscore the complementary potential of spring and winter accessions for breeding programmes. The contrasting traits between spring and winter groups highlight opportunities for targeted breeding focus, developing varieties tailored to different environmental conditions and consumer demands.

### Convarieties demonstrate distinct performances across various traits

**Fig. 5:**
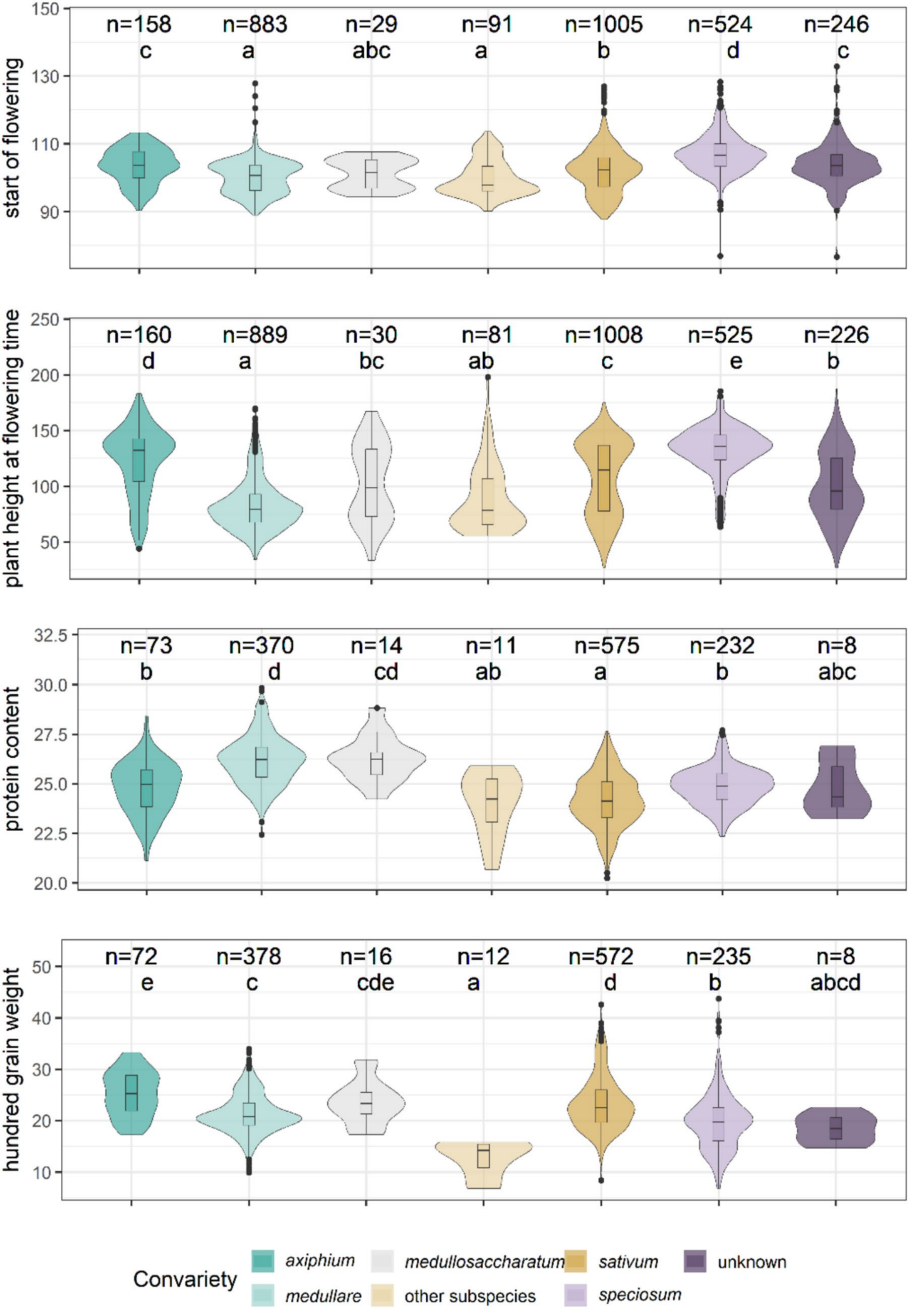
Distribution of BLUEs for start of flowering (days), plant height (cm), protein content (%), and hundred grain weight (g) across convarieties in the spring population. The numbers of BLUEs are given above the plots along with the compact letter display indicating the significance groups. The absence of overlapping letters indicate significant differences (*p* < 0.05) between convarieties

In the spring population, the analysed traits revealed varying levels of differences within and across convarieties (Fig. 5). Restrained variation in FTS among convarieties points to a consistent optimal flowering time window within the spring population. In contrast, plant height at flowering (PHFT) and HGW exhibited pronounced variability both within and across convarieties, with the widest ranges observed in the *sativum*, *speciosum* and unknown accessions. Prot also varied significantly, with *sativum* and *speciosum* convarieties achieving the highest mean values. The results consistently point to a clear association between convarietal classification and trait performance, despite unequal sample sizes across groups, emphasizing the relevance of taxonomic distinctions representing phenotypic differentiation within the spring pea population.

### Convariety-dependent trait associations inform selection strategies within pea collection

**Fig. 6:**
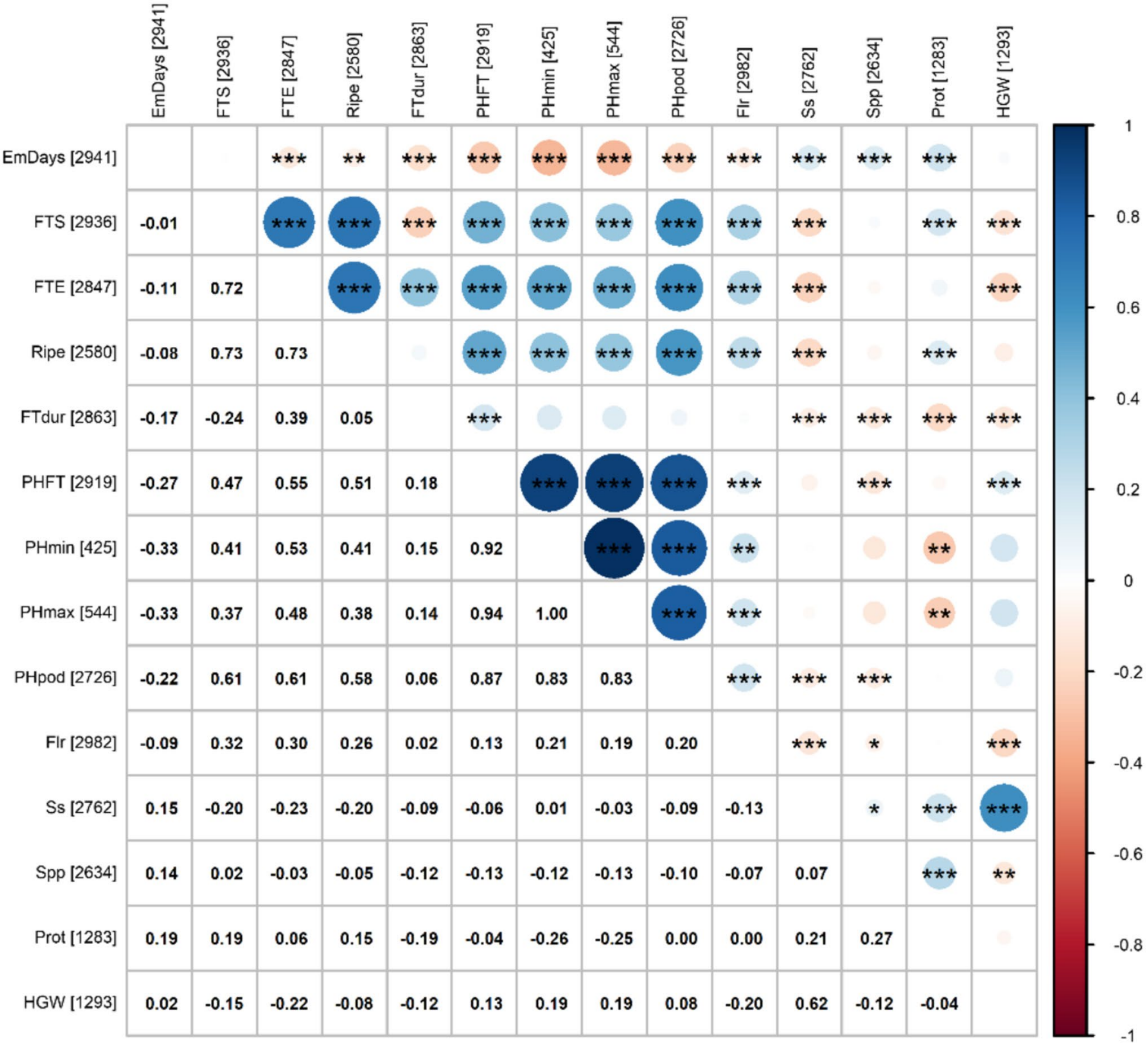
Trait correlations in the spring pea population based on the estimated BLUEs. Spearman’s correlation coefficients between traits are shown below the diagonal; corresponding visualizations using coloured symbols are shown above. Numbers in parentheses indicate the number of BLUEs available for each trait. Asterisks denote significance after Holm’s multiple testing correction (*p* < 0.05, *p* < 0.01, *p*** < **0.001). Colour and size of the circles reflect the strength and direction of the correlation, with blue indicating positive and red negative associations. EmDays = days to emergence (days since March 1th), FTS = start of flowering (days since March 1th), FTE = end of flowering (days since March 1th), Ripe = date of picking ripeness (days since March 1th), FTdur = duration of flowering (days since March 1th), PHFT = plant height at flowering time (cm), PHmin = minimum plant height (cm), PHmax = maximum plant height (cm), PHpod = height of the first pod (cm), Flr = number of flowers per inflorescence, Ss = seed size, Spp = number of seeds per pod, Prot = protein content (%), HGW = hundred grain weight (g)

Trait correlation analysis within the spring population revealed several significant associations, particularly among plant height and flowering-related traits, which appeared tightly interconnected. In contrast, HGW, Spp (number of seeds per pod), and Prot showed weaker correlations with other traits, indicating more independent performances (Fig. 6). Inspection of correlations per convarieties highlighted distinct trends, suggesting different genetic mechanisms underlying the observed trait associations. For instance, in *axiphium*, Prot showed positive links with Ss (seed size) and HGW, but negative associations with FTdur (duration of flowering), hinting at the potential to combine large seeds, high protein, and a concentrated flowering period in this convariety. In contrast, *medullare* and *sativum* exhibited positive correlations between Prot and traits related to vegetative growth and earliness, while *speciosum* displayed a trade-off between seed size/weight and Spp (Online Resource 9). In the winter population, PWSurv showed a significant positive association with PHmax (maximum plant height), while other trends, including a potential trade-off with HGW, were less evident, likely reflecting the limitations imposed by small sample size (Online Resource 10). Moreover, significant association between plant height and flowering traits further reflected coordinated developmental dynamics between these traits. These findings underscore the genetic complexity governing the interplay of these important agronomic characteristics in pea, identifying the variation and the potential within the IPK pea populations to capitalize on by breeders.

## Discussion

Unlocking the valuable, yet largely untapped, diversity within genetic resources is crucial for breeding efforts targeting the challenges posed by increasingly harsh environmental conditions. This need sparked an intensified interest in the curation and consolidation of historical phenotypic data to drive the transition from genebanks into bio-digital resource centres (Mascher et al. 2019). Building on the success of these previous efforts that focused primarily on wheat (Philipp et al. 2018; Svoboda et al. 2024) and barley (El Hanafi et al. 2023; González et al. 2018), we were motivated to unlock historical phenotypic data collected during regeneration for the crop species pea, using the IPK Gatersleben collection as a case study, which is the largest in the EU (Smýkal et al. 2012).

### Selective outlier control enables robust insights from historical data

In line with previous studies (Berkner et al. 2024b; Bernal-Vasquez et al. 2016; El Hanafi et al. 2023; González et al. 2018; Philipp et al. 2018; Svoboda et al. 2024), our findings demonstrated that rigorous plausibility checks and systematic outlier removal substantially enhanced data quality as reflected by the increased heritability estimates across traits. Dependence on historical non-orthogonal data entails the risk of inaccurate extrapolation to contemporary conditions, especially when genotype-by-environment interactions are demonstrated (Dawson et al. 2013; He et al. 2016). However, in wheat high correlations were found between BLUEs obtained from historical and validation data (Philipp et al. 2018). We addressed the inherent challenge of non-orthogonality by employing linear mixed models that anchored year effects on frequently phenotyped accessions. This improved the accuracy of trait estimates for sparsely tested entries increasing the number of accessions with reliable phenotypic information (González et al. 2018; Philipp et al. 2018). In contrast to normalized rank products (Keilwagen et al. 2014), which rescale values to within-year ranks and omit true variance partitioning, and generic robust estimators (Morato and Stojanović 2021) that uniformly down-weight all outliers our pipeline combines trait-specific plausibility thresholds with mixed-model residual diagnostics. This hybrid strategy maintains proper extreme observations, which is critical to capturing seasonal or experimental outliers, while firmly restraining residual variance under the unreplicated and unbalanced data. Looking forward, this framework can be extended with cross-validation to fine-tune threshold settings or with hierarchical Bayesian outlier models that estimate error probabilities at observation level. In such way, we shift the phenotypic curation from a one-time task into a continuous data-driven calibration that steadily enhances heritability estimates and sets a new standard for genebank phenomics.

### The diverse collection of IPK pea accessions inspires targeted breeding for various ideotypes

A diverse germplasm base is essential for unravelling complex trait architecture and driving breeding innovation. The IPK pea collection spans accessions from hot desert to subarctic climates (Köppen-Geiger classification; Peel et al. 2007), reflecting a wide ecological amplitude with potential implications for agronomic trait expression. The curated phenotypic data revealed that IPK retains exceptionally broad variation in key agronomic traits, demonstrating six-fold differences in grain weight, a 56 day span in flowering time, and over 29.8% protein content among genebank accessions that go well beyond simple geographic or taxonomic structuring. For breeding purposes, this diversity can offer a powerful toolkit to match ideotypes adapted to varied climate requirements and markets demands (Carlson-Nilsson et al. 2021; Karkanis et al. 2016; Kuzbakova et al. 2022; Rubiales et al. 2021b). For instance, earliness confers dual benefits by mitigating terminal drought stress and reducing seed loss due to pod shattering, making it a key target for ideotype design in both temperate and marginal environments (Anderson 1955; Funatsuki et al. 2014; Lush et al. 1980; Parker et al. 2021). Moreover, improving protein content in pea is one of the central breeding objectives (Krefting 2017; Shanthakumar et al. 2022; Tao et al. 2017; Yang et al. 2023), to power its role in the protein transition as a key alternative to soybean in temperate regions (Krefting 2017; Shanthakumar et al. 2022). Simultaneously, combining protein content above 25% (Daba and Morris 2022; Yang et al. 2023) with hundred-seed weight over 32 g defines a high-value trait package that breeders can leverage for developing pea varieties with quality-driven breeding targets (Bundessortenamt 2024; Marton Genetics 2023; Processors and Growers Research Organisation 2025).

The distinct trait correlations observed within each convariety underscore the value of moving beyond global core-sets to convariety-specific selection indices (Chung and Liao 2022; Maranna et al. 2021; Najafi et al. 2024; Ouattara et al. 2024). Reframing ideotype performance as selection indices allows design of ideotype-specific core collections (Olivoto and Nardino 2020). On trait level, tailored core collections demonstrated increased statistical power, although the trade-off in increased population structure has to be managed (Berkner et al. 2024a). By maximizing diversity for the trait set shaping the ideotype, investigation of mechanisms defining crop suitability for key roles can be accelerated (Ravasi et al. 2020; Rocha et al. 2018). To ensure these sub-collections also perform in target environments, they can be further refined with the focused identification of germplasm strategy (FIGS, Khazaei et al. 2013), which uses environmental metadata to select accessions most likely to express the desired trait combinations under specific agroecological conditions. This two-step framework yields compact, high-resolution germplasm sets ideally suited for efficient pre-breeding.

### Promising frost tolerance in IPK winter pea collection

The transition from spring to winter pea cultivation is key to mitigate spring and summer drought stress, an increasingly pressing issue under climate change, by permitting early root development and maturity. Furthermore, winter peas also offer notable advantages, including increased yield and enhanced soil benefits (Bagheri et al. 2023; Neugschwandtner et al. 2020; Urbatzka et al. 2012). However, the expansion of winter pea cultivation depends on the availability of consistently winter-hardy material (Homer et al. 2016; Karkanis et al. 2016). Success requires avoiding winter‐kill while ensuring timely and vigorous spring growth. The historical winter data showed that *speciosum* whose flowering begins latest among winter types generally also achieved the highest post-winter survival (> 79%, *r* = 0.55), whereas lines that flower earlier average below 50% survival. This suggests that delaying reproductive onset until after peak cold periods shields meristems from lethal freezes (Klein et al. 2014; Maqbool et al. 2010). Furthermore, the acceleration of growth and development preceding reproductive onset is associated to irreversible deacclimation, increasing the importance of strict regulation of these developmental progresses via, e.g. photoperiod sensitivity (Leinonen et al. 1997; Lejeune-Hénaut et al. 2008). These phenology/survival dynamics exhibit a Heritability of 0.84, indicating strong genetic control and reliable repeatability across divergent frost regimes.

Records of the IPK winter pea population cultivated in spring without adverse phenotypical effects indicate their possibly facultative identity, which would enable short breeding cycles and winter cultivation in regions with unreliable frost (Bauerle 2019). Moreover, occasional overwintering rates above 100% where new seedlings emerge during winter have been documented in peas and other crops and are attributed to cycling between primary physical dormancy and environmentally induced secondary dormancy (Batlla and Benech-Arnold 2006; Short et al. 2003; Urbatzka et al. 2011). In our data, unfavourable moisture and temperature fluctuations may similarly delay or re-impose dormancy, effectively postponing emergence until conditions become safe. While we lack direct dormancy measures, this dormancy–phenology coupling likely underpins the observed survival advantages and offers an additional, underexploited axis for breeding truly winter‐hardy pea cultivars.

### Towards an integrative approach for pea breeding

Recent EU Common Agricultural Policy and Rural Development Plan subsidies aim to expand legume acreage and meet the European Green Deal’s “Farm to Fork” goals, yet pulses, including pea, still lag behind cereals due to lower yields, unstable production, and underinvestment in R&D (Magrini et al. 2016; Murphy-Bokern 2022; Rubiales et al. 2021a). Therefore, breeding pea must embrace integrative strategies that capitalize on both data-driven insights and cutting-edge technology. Historical data reveal phenotypic plasticity across decades and diverse climates. Leveraging this information enables the identification of accessions adapted to specific agroecological niches, guiding the design of next-generation ideotypes (Alseekh et al. 2025). Yet full-scale phenotyping remains costly and time-intensive, leaving substantial portions of the collection only sparsely characterized. Cost-effective high-throughput genotyping offers a pragmatic workaround that can safeguard genetic integrity by flagging allele-frequency shifts that signal drift during regeneration and furnish the dense marker panels needed for downstream analyses. Although the large and repetitive pea genome long impeded genomic research, targeted core collections from USDA and European genebanks have nonetheless enabled GWAS to uncover major-effect loci albeit with limited mapping resolution due to sparse SNP coverage (Burstin et al. 2007; Crosta et al. 2023; Irzykowska and Wolko 2004; Klein et al. 2020). In parallel, genomic prediction has emerged as a powerful extension of genome wide-association studies (GWAS), capable of aggregating small‐effect loci to forecast complex traits (Goddard 2009; Meuwissen et al. 2001). However, the effectiveness of these predictions can be compromised when applied to populations with limited genetic diversity or when environmental conditions differ significantly from those encountered during model training (Wientjes et al. 2015). This highlights the need for ongoing refinement of prediction methodologies to ensure robust predictions across diverse breeding scenarios (Bari et al. 2021; Crosta et al. 2023; Tayeh et al. 2015). Across-genebank predictions can address this limitation by training on a broader spectrum of allelic diversity and environmental contexts. For instance, models built on one repository still achieved moderate predictive power when tested in another, despite differences in allele frequencies and trial protocols (El Hanafi et al. 2023; Schulthess et al. 2022). Therefore, unlocking the phenotype data of the IPK genebank collection, anticipating a future genotyping endeavour similar to the USDA and JIC UK (Cheng et al. 2015; Jing et al. 2010), is valuable not only for the collection itself, but once combined with genotype data, also provides a resource to characterize other collections. By pooling marker data, trial metadata, and phenotypes across multiple collections, genebank curators can mitigate both genetic-base and environment-shift limitations, allowing them to impute unphenotyped traits (e.g. frost tolerance, disease resistance) in sparsely characterized accessions without additional field trials. Integrating such across-genebank predictions with machine‐learning algorithms that prioritize haplotype-trait associations further enables creation of compact, high-resolution training sets tailored to specific breeding objectives.

## Supplementary Information

Below is the link to the electronic supplementary material.

Supplementary file1 (DOCX 2413 kb)

## Data Availability

A data publication will become available shortly. In the meantime, data can be requested from the corresponding author.
