# Supplementary material for: Harnessing historical genebank data to accelerate pea breeding
Source: Theor Appl Genet. 2025 Sep 9;138(10):243. doi: 10.1007/s00122-025-05032-5 (PMC12420702; doi:10.1007/s00122-025-05032-5)

**Online Resource 1** Number of pea accessions of the IPK genebank collected in each country of provenance indicated in ISO Alpha-3 code format, for spring and winter pea.

| **Country of provenance** | **Type** | **Nr. of accessions** |  | **Country of provenance** | **Type** | **Nr. of accessions** |
| --- | --- | --- | --- | --- | --- | --- |
| AFG | Spring | 88 |  | LBN | Spring | 2 |
| ALB | Spring | 9 |  | LBY | Spring | 5 |
| ARM | Spring | 2 |  | LTU | Spring | 1 |
| AUS | Spring | 7 |  | LVA | Spring | 7 |
| AUT | Spring | 4 |  | MAR | Spring | 1 |
| BDI | Spring | 4 |  | MEX | Spring | 1 |
| BEL | Spring | 2 |  | MKD | Spring | 2 |
| BGR | Spring | 39 |  | MNG | Spring | 12 |
| BLR | Spring | 1 |  | NLD | Spring | 198 |
| BOL | Spring | 1 |  | NPL | Spring | 2 |
| BRA | Spring | 1 |  | NZL | Spring | 1 |
| BTN | Spring | 10 |  | OMN | Spring | 4 |
| CAN | Spring | 12 |  | PER | Spring | 2 |
| CHE | Spring | 2 |  | POL | Spring | 53 |
| CHL | Spring | 1 |  | PRK | Spring | 3 |
| CHN | Spring | 35 |  | PRT | Spring | 6 |
| COD | Spring | 15 |  | ROU | Spring | 9 |
| COL | Spring | 1 |  | RUS | Spring | 40 |
| CSK | Spring | 33 |  | RWA | Spring | 11 |
| CUB | Spring | 3 |  | SDN | Spring | 4 |
| CZE | Spring | 2 |  | SUN | Spring | 147 |
| DEU | Spring | 599 |  | SVK | Spring | 65 |
| DNK | Spring | 15 |  | SWE | Spring | 46 |
| DZA | Spring | 13 |  | SYR | Spring | 8 |
| EGY | Spring | 4 |  | TJK | Spring | 2 |
| ESP | Spring | 35 |  | TUN | Spring | 16 |
| EST | Spring | 2 |  | TUR | Spring | 16 |
| ETH | Spring | 115 |  | UKR | Spring | 12 |
| FIN | Spring | 11 |  | USA | Spring | 117 |
| FRA | Spring | 155 |  | UZB | Spring | 5 |
| GBR | Spring | 101 |  | YEM | Spring | 30 |
| GEO | Spring | 46 |  | YUG | Spring | 5 |
| GRC | Spring | 58 |  | ZAF | Spring | 3 |
| HRV | Spring | 8 |  | ZMB | Spring | 7 |
| HUN | Spring | 25 |  | ALB | Winter | 5 |
| IND | Spring | 11 |  | BGR | Winter | 1 |
| IRN | Spring | 1 |  | CHN | Winter | 2 |
| IRQ | Spring | 1 |  | DEU | Winter | 15 |
| ISR | Spring | 10 |  | FRA | Winter | 2 |
| ITA | Spring | 77 |  | GRC | Winter | 19 |
| JPN | Spring | 3 |  | SUN | Winter | 1 |
| KAZ | Spring | 1 |  |  |  |  |

**Online Resource 2** Number of total and unique records per decade for each of the traits in spring (a) and winter (b) accessions. Above each bar the total number of records and unique records are summarized. Days are measured since March 1^th^ for spring- and January 1^th^ for winter accessions. EmDays = days to emergence, FTS = start of flowering (days), FTE = end of flowering (days), Ripe = date of picking ripeness (days), FTdur = duration of flowering (days), PHFT = plant height at flowering time (cm), PHmin = minimum plant height (cm), PHmax = maximum plant height (cm), PHpod = height of the first pod (cm), Flr = number of flowers per inflorescence, Ss = seed size, Spp = number of seeds per pod, Prot = protein content (%), HGW = hundred grain weight (g), Surv = survival rate (%), PWSurv = post-winter survival rate (%).


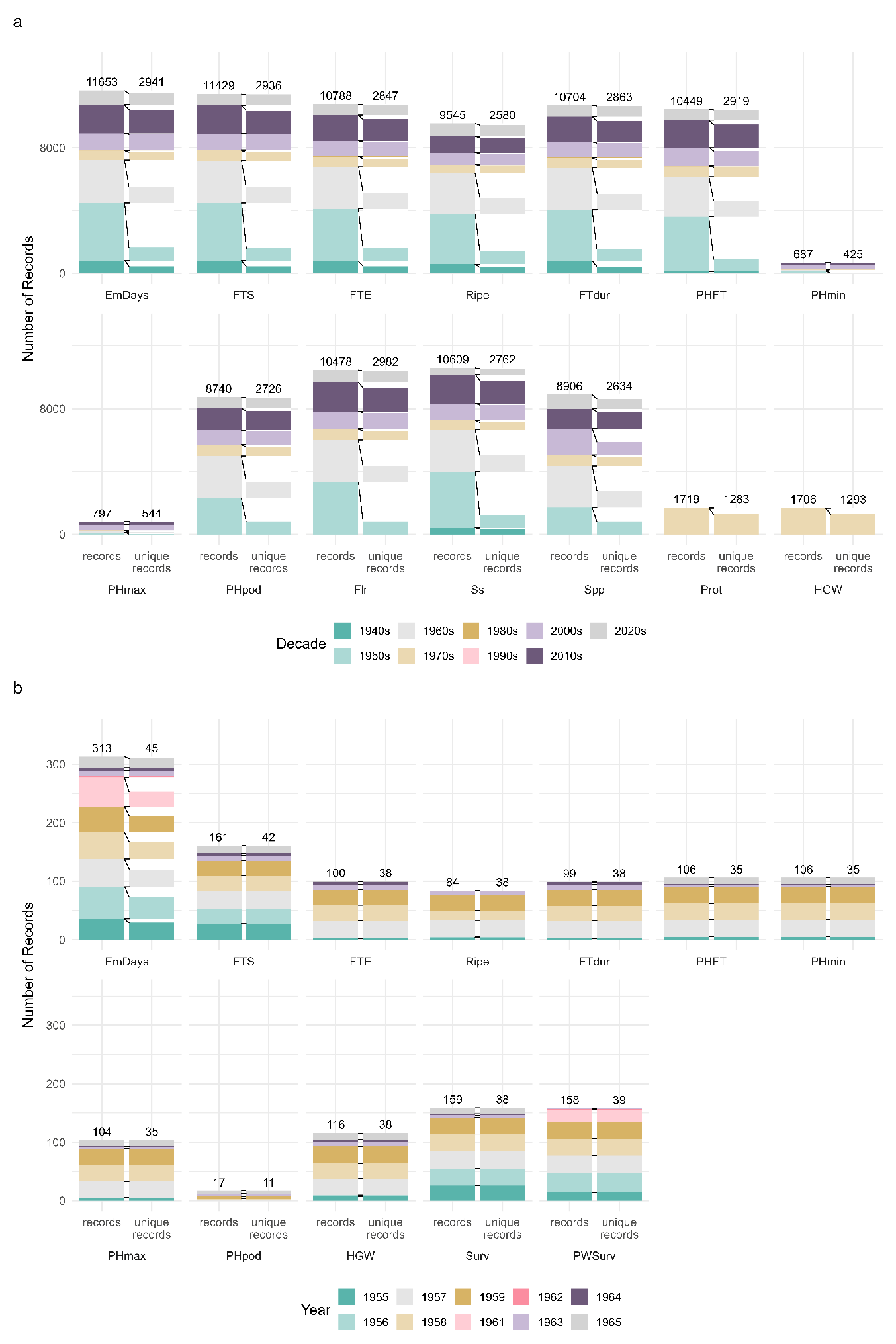


**Online Resource 3** Estimated year effects on the start and end of flowering and the number of seeds per pod for spring accessions. Grey lines indicate the upper- and lower boundaries corresponding to an absolute CV of 3, above and below which classification of years as outliers was assigned. Outliers are visualized in dark.


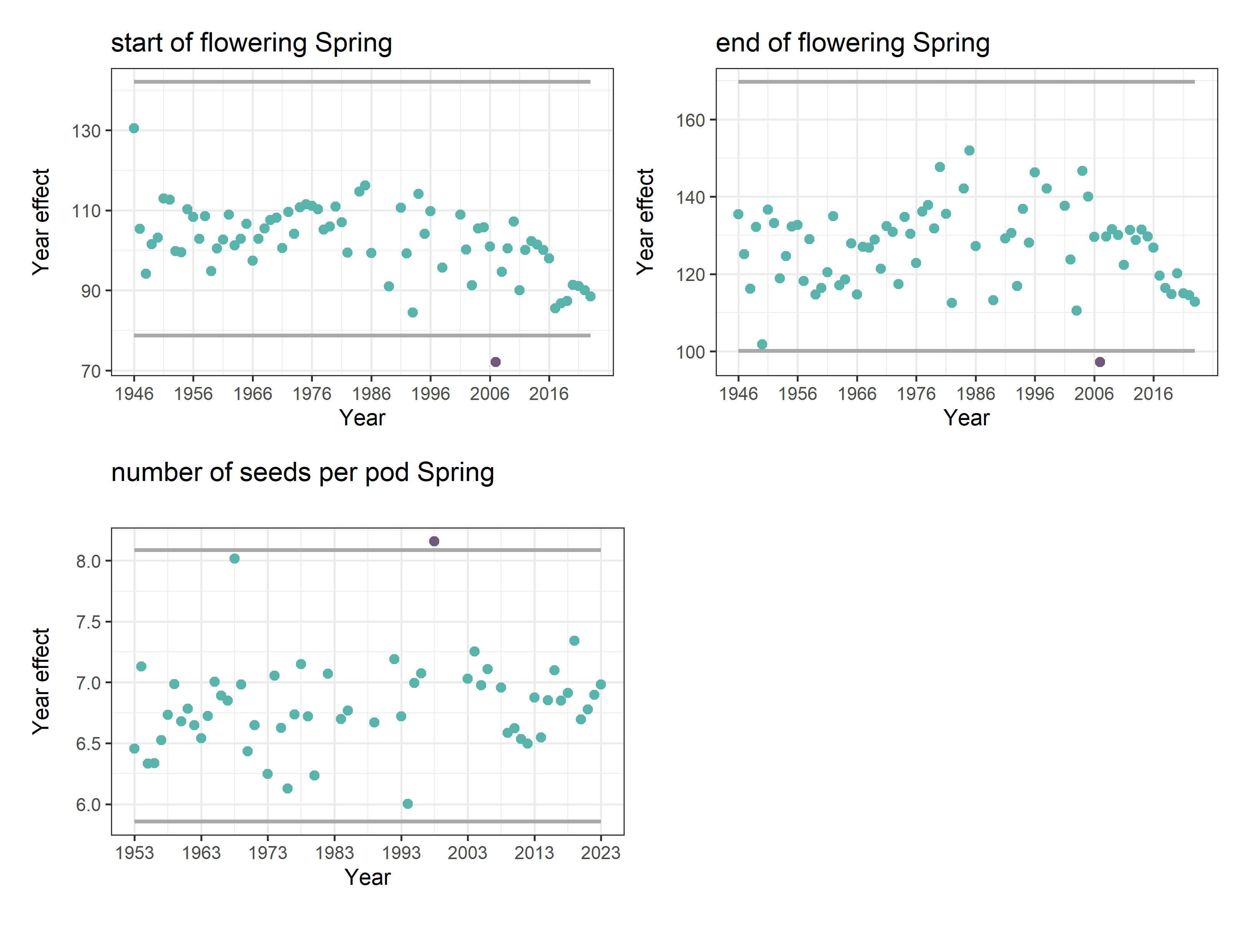


**Online Resource 4** Studentized residuals showing the distribution of observations marked as outliers throughout the regeneration years for the traits start of flowering, duration of flowering, plant height at flowering time, hundred grain weight, and for spring accessions number of seeds per pod and protein content and for winter accessions days to emergence and post-winter survival. Each alternation of colour indicates a different year, and the length on the x-axis gives an impression of the number of observations relative to other years for the same trait. Some years, of which no observations were available for the certain trait, were omitted from the figure. Observations with a studentized residual that exceeded the boundary of a Tukey test based on all studentized residuals for that trait were considered as outliers. The boundary is visualized as dashed lines.


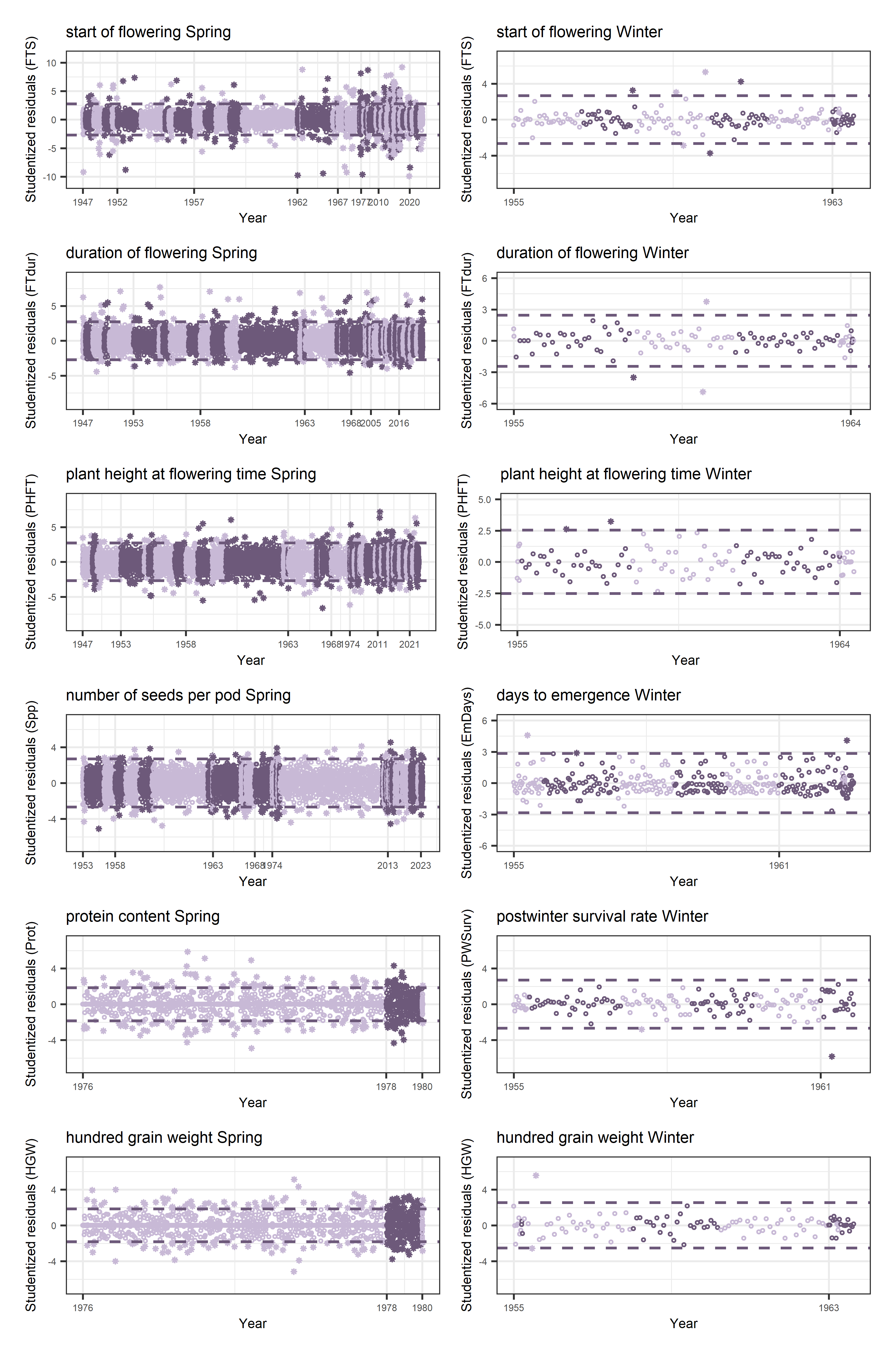


**Online Resource 5** Variance components that supported calculation of the final heritability. * P ≤ 0.05, ** P ≤ 0.01, *** P ≤ 0.001, ^NS^ = not significant

| Trait |  | $\sigma_{G}^{2}$ | $\sigma_{E}^{2}$ | $\sigma_{GxE}^{2}$ | $\sigma_{e}^{2}$ | $\sigma_{C}^{2}$ |
| --- | --- | --- | --- | --- | --- | --- |
| Days to emergence | Spring | 5.69^***^ | 75.21^***^ | 3.11^***^ | 0.17 | 0.09^***^ |
|  | Winter | 0.00 ^NS^ | 1.89^***^ | 0.00 ^NS^ | 12.84 | - |
| Start of flowering | Spring | 36.34^***^ | 60.50^***^ | 3.13^*^ | 2.74 | 7.48^***^ |
|  | Winter | 11.65^***^ | 30.78^***^ | - | 5.61 | 52.73^***^ |
| End of flowering | Spring | 38.38^***^ | 95.63^***^ | 9.68^***^ | 7.22 | 13.07^***^ |
|  | Winter | 2.42 ^NS^ | 129.59^***^ | - | 11.03 | 49.98^**^ |
| Date of picking ripeness | Spring | 29.85^***^ | 91.91^***^ | 0.00 ^NS^ | 11.66 | 9.79^***^ |
|  | Winter | 0.00 ^NS^ | 209.19^***^ | - | 1.87 | 65.12^***^ |
| Duration of flowering | Spring | 11.10^***^ | 40.16^***^ | 12.05^***^ | 8.75 | 1.20^***^ |
|  | Winter | 1.47 ^NS^ | 92.94^***^ | - | 11.82 | - |
| Plant height at flowering time | Spring | 832.27^***^ | 311.67^***^ | 105.40^***^ | 49.24 | 366.65^***^ |
|  | Winter | 146.05^***^ | 185.74^***^ | - | 119.05 | 325.56^**^ |
| Plant height minimal | Spring | 839.23^***^ | 387.25^***^ | - | 63.71 | 589.65^***^ |
|  | Winter | 227.65^***^ | 127.42^***^ | - | 158.44 | - |
| Plant height maximal | Spring | 308.22^***^ | 92.07^***^ | 0.00 ^NS^ | 103.66 | 142.09^***^ |
|  | Winter | 206.97^***^ | 353.72^***^ | - | 152.83 | 611.31^**^ |
| Height of the first pod | Spring | 308.22^***^ | 92.07^***^ | 0.00 ^NS^ | 103.66 | 142.09^***^ |
|  | Winter | 328.04^**^ | 0.00 ^NS^ | - | 28.23 | - |
| Number of flowers per inflorescence | Spring | 0.29^***^ | 0.04^***^ | 0.00 ^NS^ | 0.18 | 0.11^***^ |
|  | Winter |  |  |  |  |  |
| Seed size | Spring | 0.18^***^ | 0.01^***^ | 0.00 ^NS^ | 0.09 | 0.14^***^ |
|  | Winter |  |  |  |  |  |
| Number of seeds per pod | Spring | 0.59^***^ | 0.06^***^ | 0.00 ^NS^ | 0.69 | 0.26^***^ |
|  | Winter |  |  |  |  |  |
| Protein content | Spring | 1.75^***^ | 2.61^***^ | 0.00 ^NS^ | 0.91 | 1.11^***^ |
|  | Winter |  |  |  |  |  |
| Hundred grain weight | Spring | 25.33^***^ | 1.56^***^ | 0.21 ^NS^ | 2.23 | 17.34^***^ |
|  | Winter | 2.91^***^ | 3.05^***^ | - | 1.30 | 6.44^*^ |
| Survival rate | Spring |  |  |  |  |  |
|  | Winter | 1.83 ^NS^ | 45.32^***^ | - | 43.86 | - |
| Post-winter survival rate | Spring |  |  |  |  |  |
|  | Winter | 135.68^***^ | 235.28^***^ | - | 74.69 | 985.14^***^ |

**Online Resource 6** The yearly count of days on which the daily minimum temperature reached below ‑14 °C under snow cover below 5 cm, visualized per winter pea accession. The numbers in brackets show the number of cultivation years per accession.


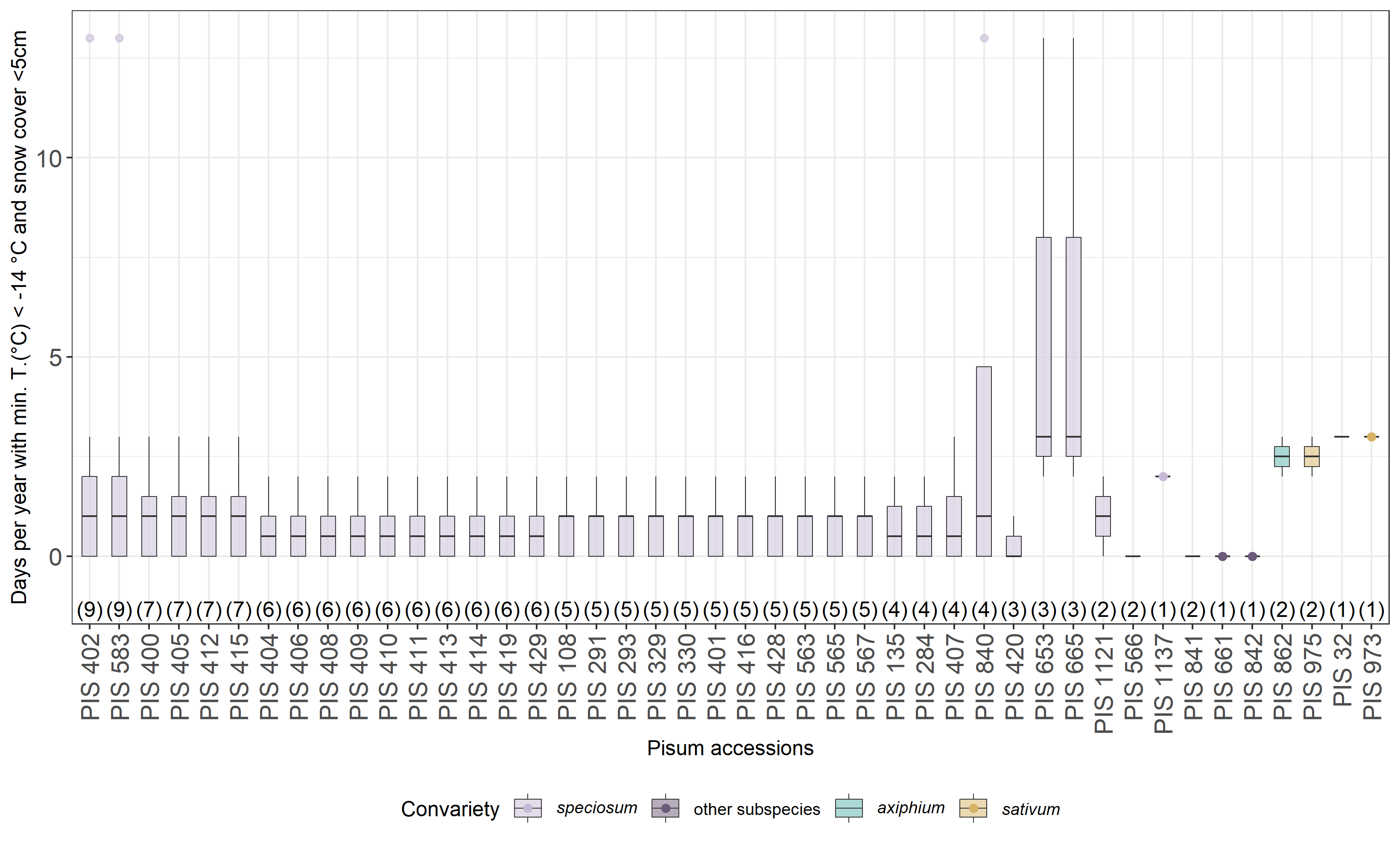


**Online Resource 7** Daily minimum temperature at 5 cm above ground (bars) and snow cover (dots) during winter pea cultivation. Temperatures below ‑6 °C under snow cover below 5 cm is defined as mild frost stress (yellow), and temperatures below ‑14 °C and under snow cover below 5 cm as severe frost stress (purple).


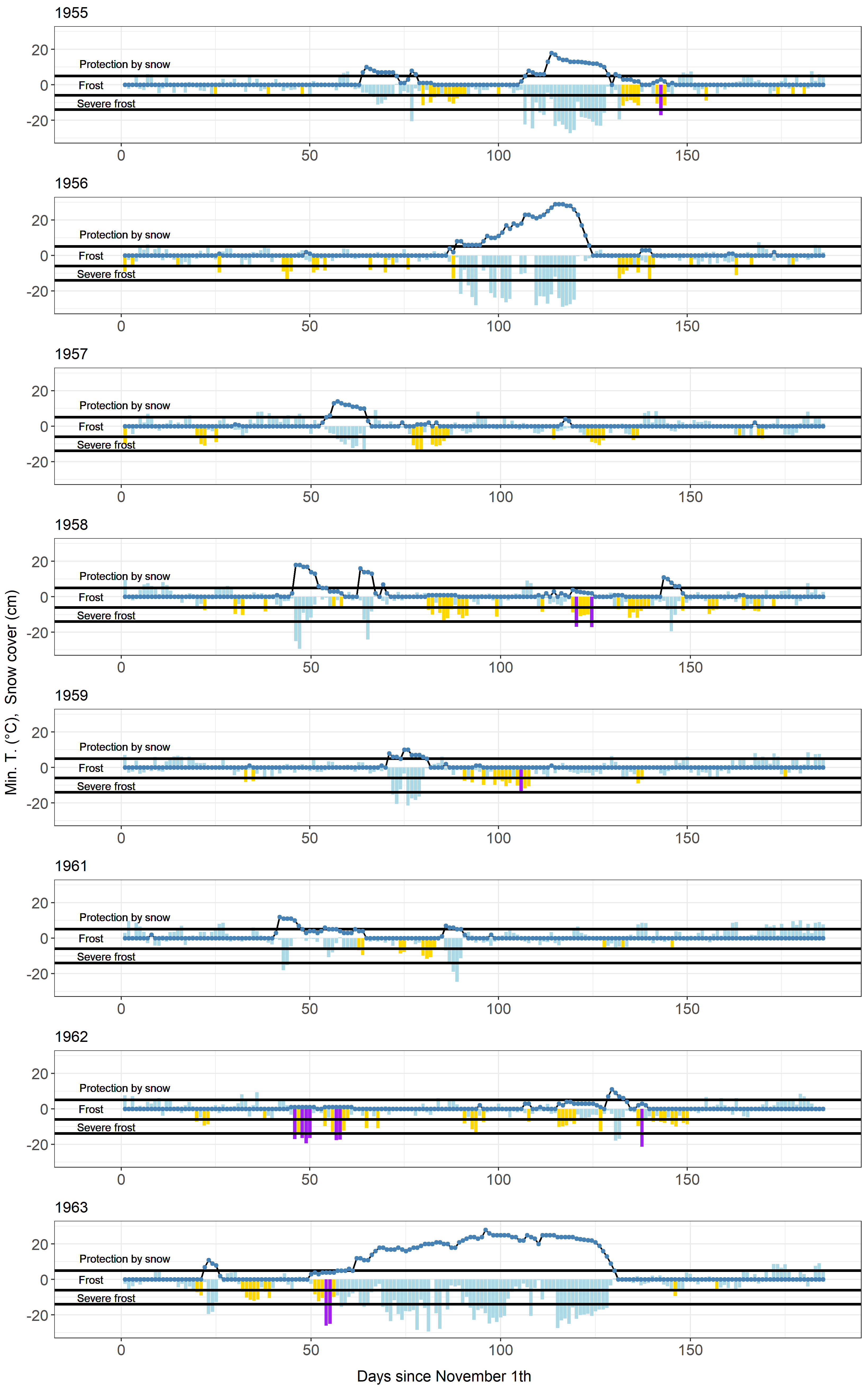


**Online Resource 8** BLUEs per trait for spring and winter accessions. The numbers of BLUEs are given above the graphs


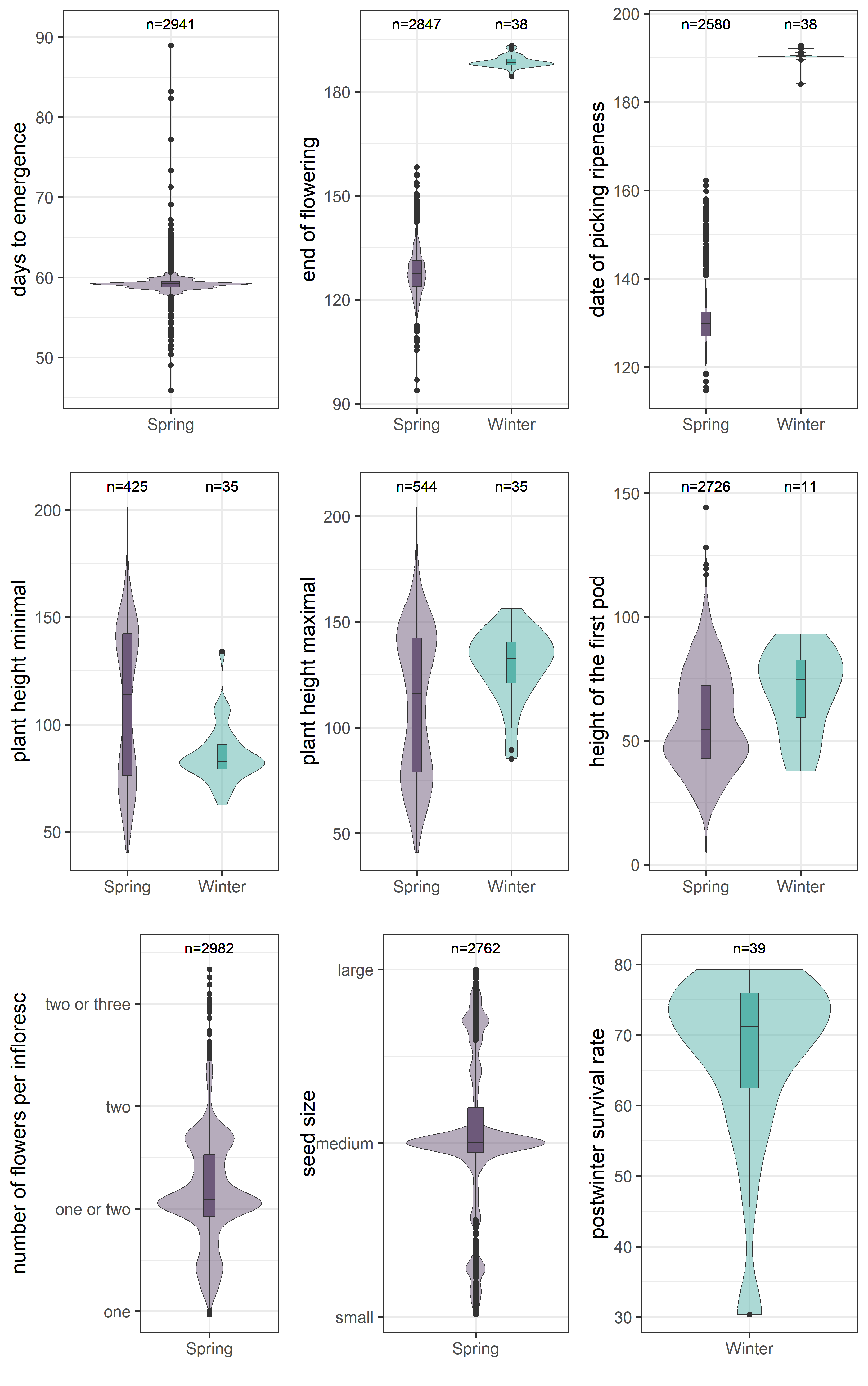


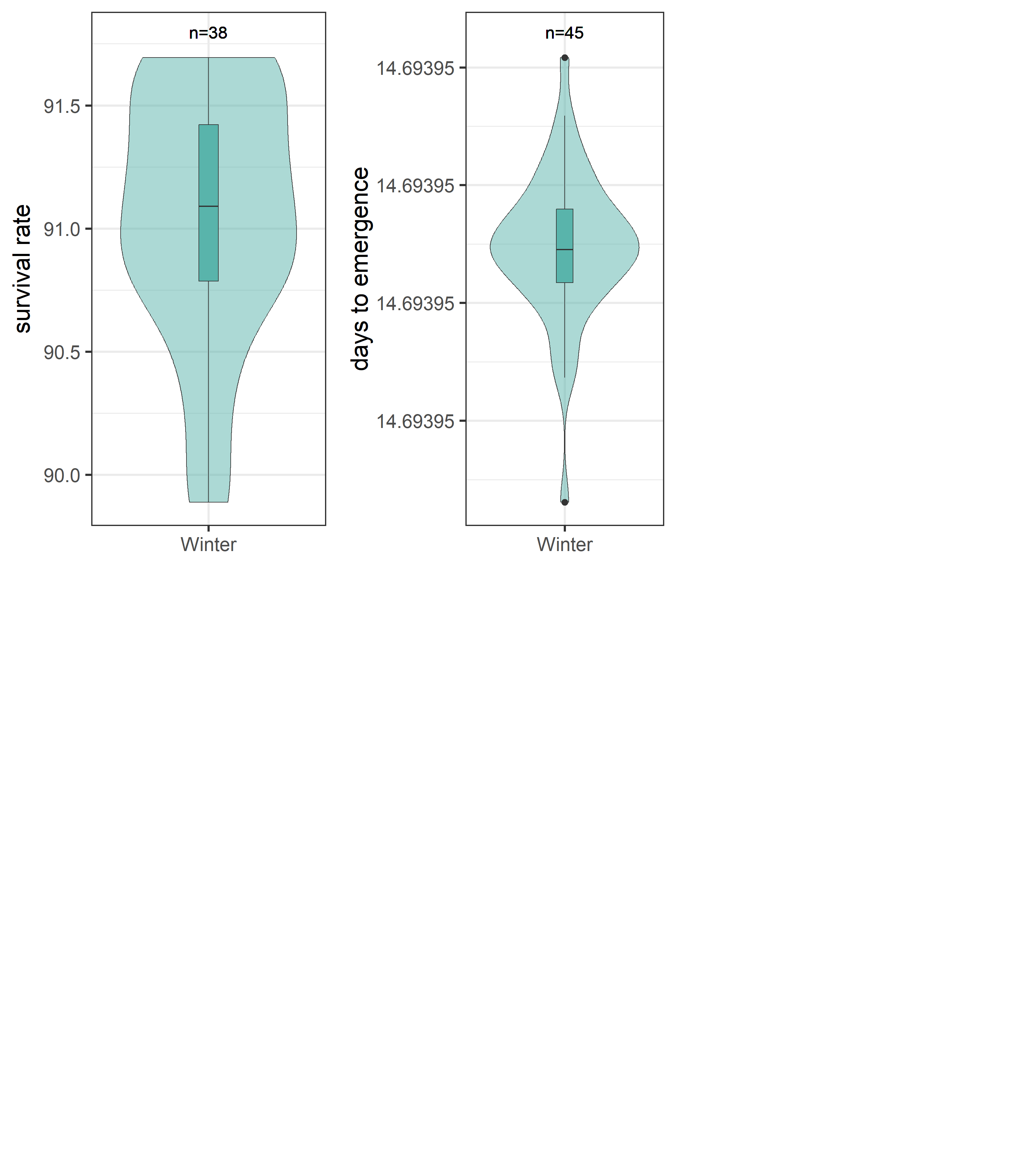


**Online Resource 9** Trait correlations in the spring pea convarieties based on the estimated BLUEs. Spearman’s correlation coefficients between traits are shown below the diagonal; corresponding visualizations using coloured symbols are shown above. Numbers in parentheses indicate the number of BLUEs available for each trait. Asterisks denote significance after Holm’s multiple testing correction (p < 0.05, **p** < 0.01, **p** < 0.001). Colour and size of the circles reflect the strength and direction of the correlation, with blue indicating positive and red negative associations. EmDays = days to emergence (days since March 1^th^), FTS = start of flowering (days since March 1^th^), FTE = end of flowering (days since March 1^th^), Ripe = date of picking ripeness (days since March 1^th^), FTdur = duration of flowering (days since March 1^th^), PHFT = plant height at flowering time (cm), PHmin = minimum plant height (cm), PHmax = maximum plant height (cm), PHpod = height of the first pod (cm), Flr = number of flowers per inflorescence, Ss = seed size, Spp = number of seeds per pod, Prot = protein content (%), HGW = hundred grain weight (g).


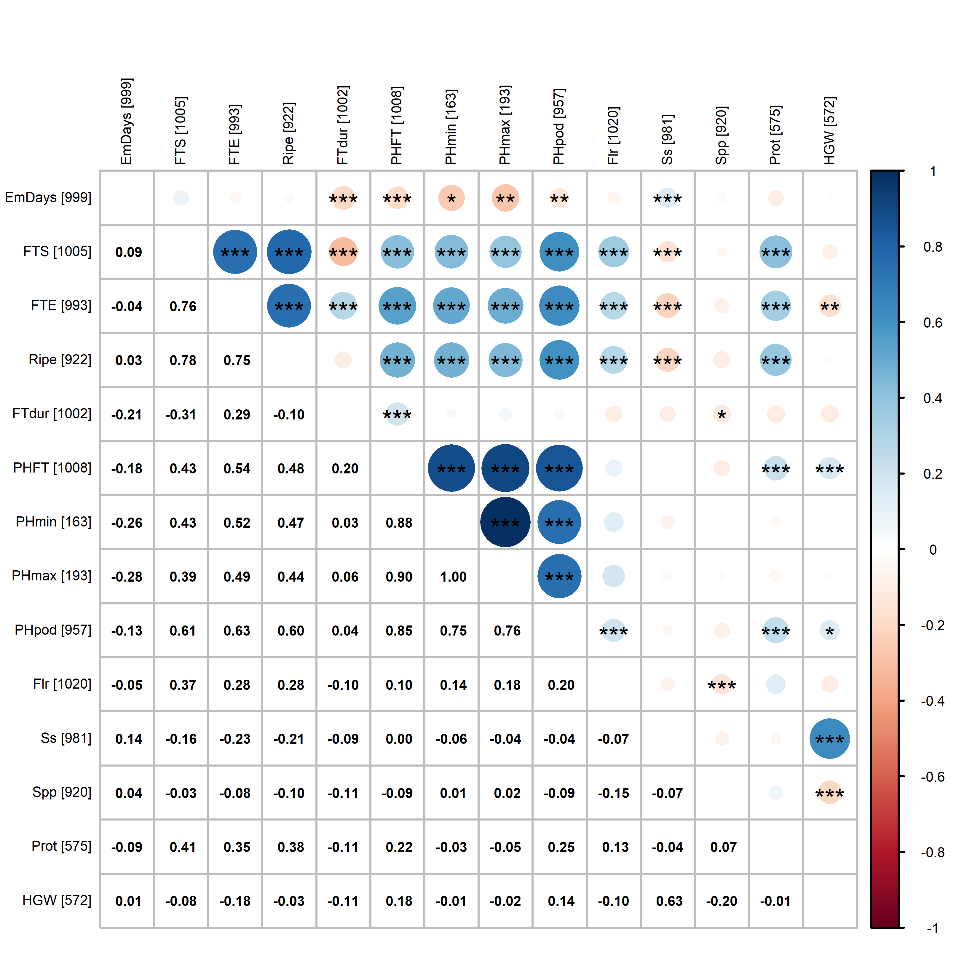


Convariety: *sativum*


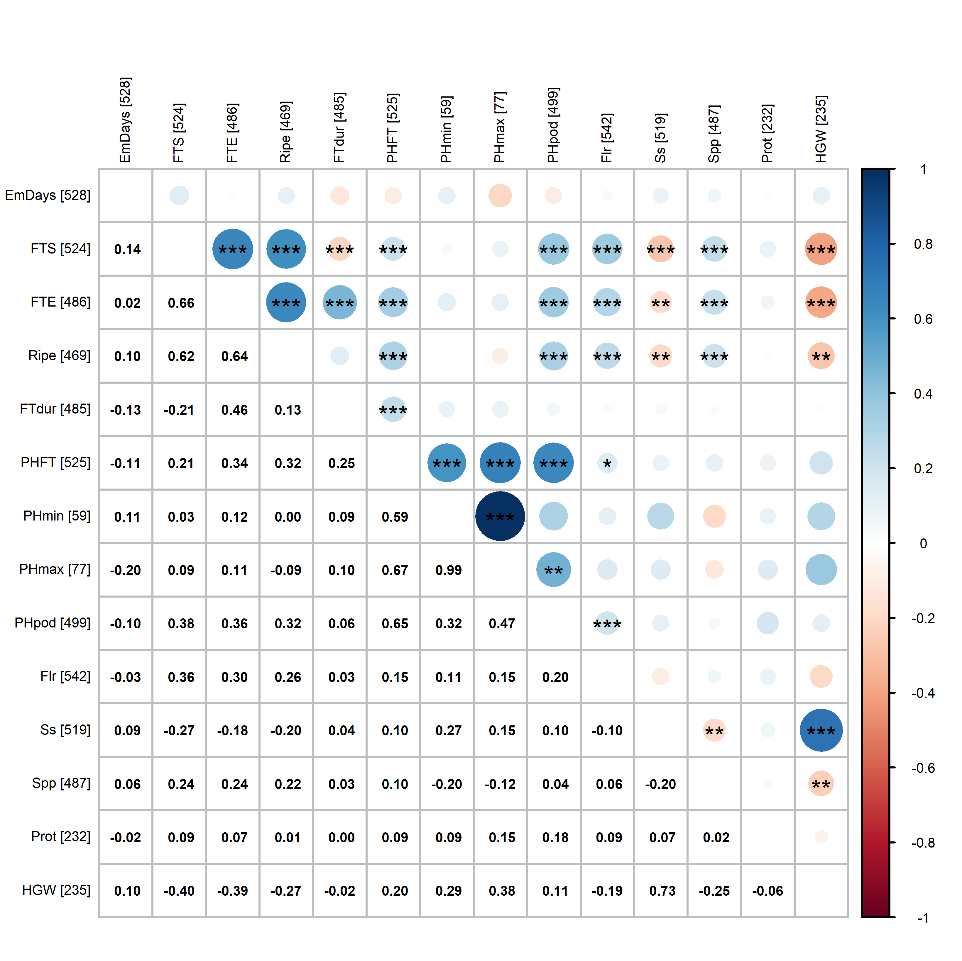


Convariety: *speciosum*


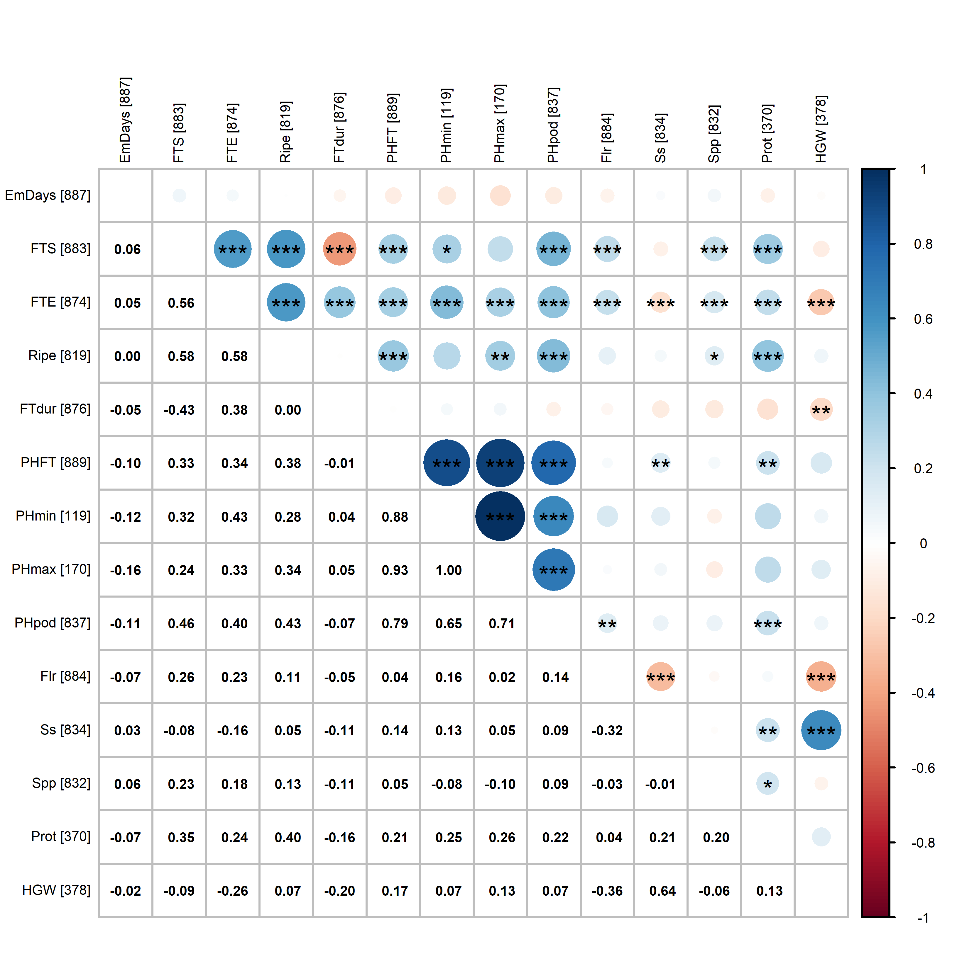


Convariety: *medullare*


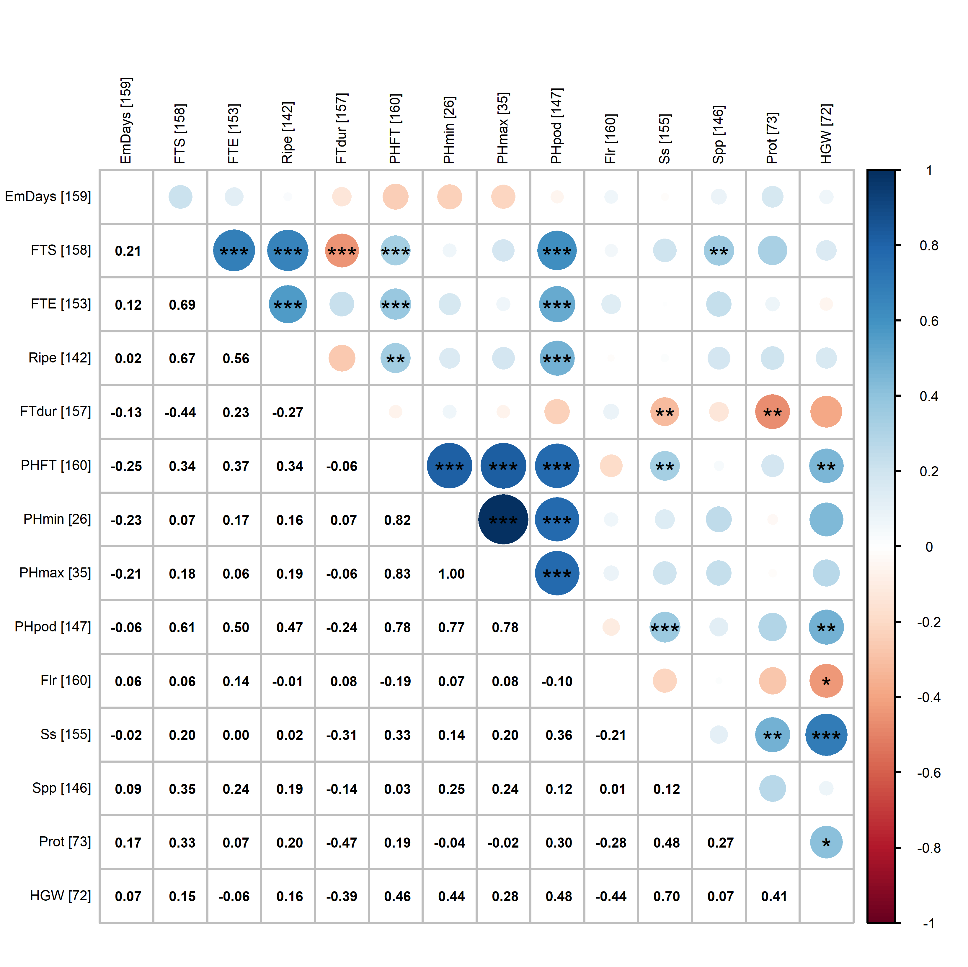


Convariety: *axiphium*


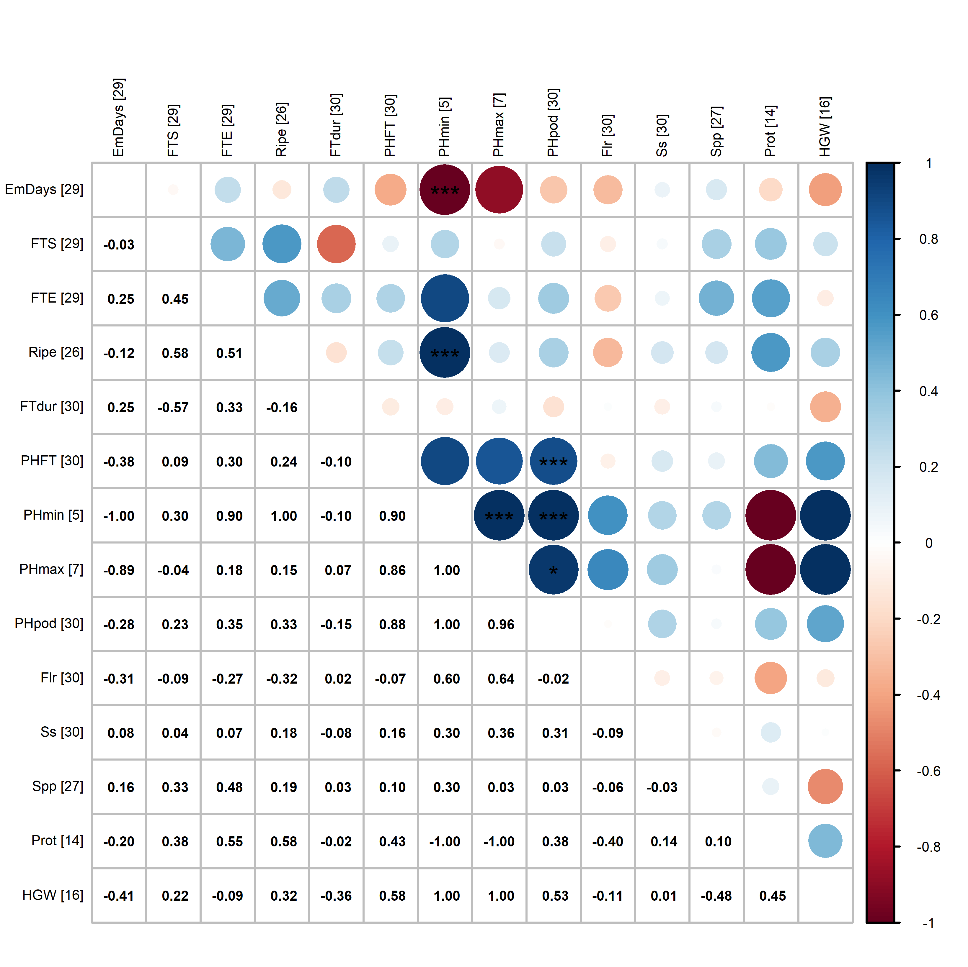


Convariety: *medullosaccharatum*


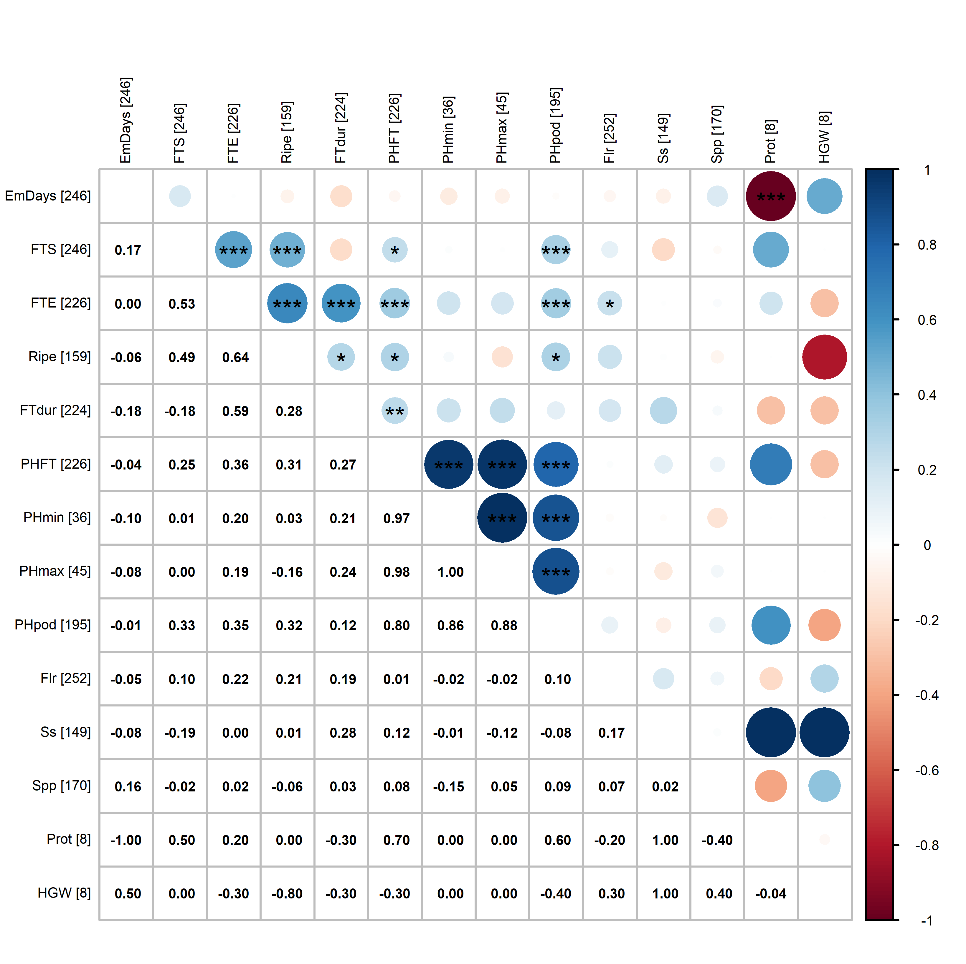


Unknown convariety


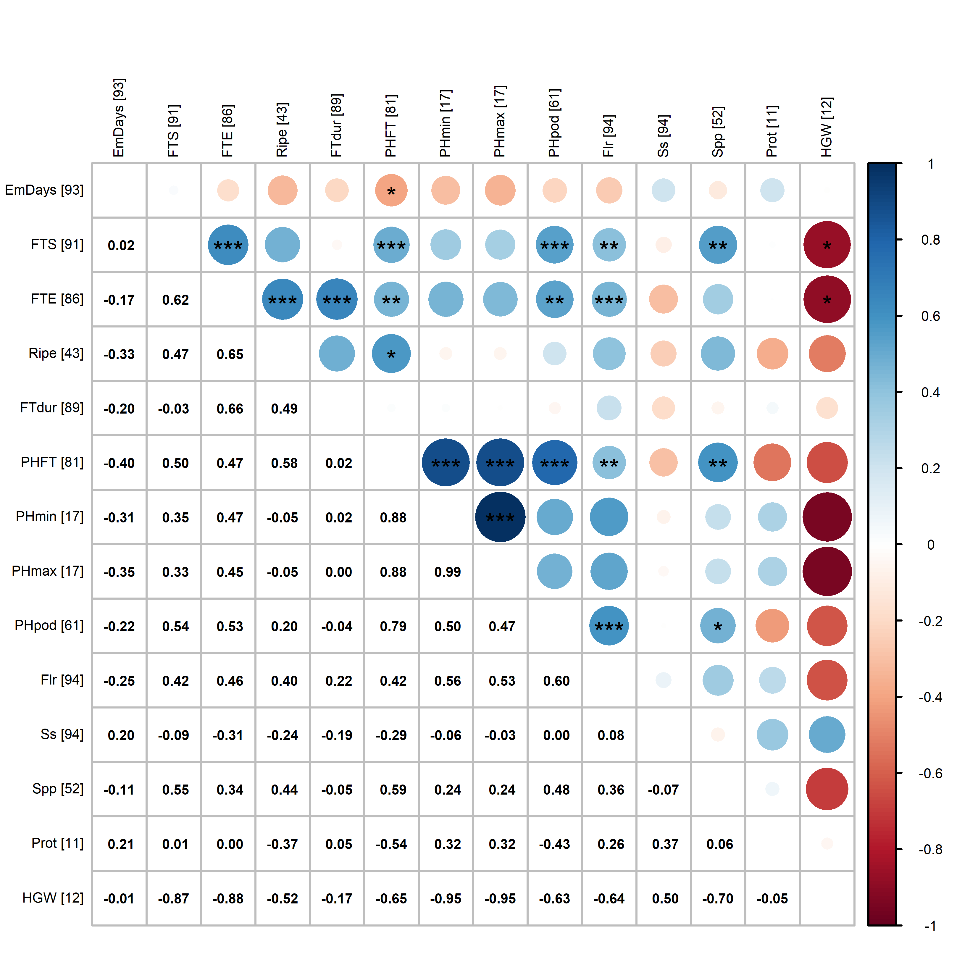


Other subspecies

**Online Resource 10** Trait correlations in the winter pea population based on the estimated BLUEs. Spearman’s correlation coefficients between traits are shown below the diagonal; corresponding visualizations using coloured symbols are shown above. Numbers in parentheses indicate the number of BLUEs available for each trait. Asterisks denote significance after Holm’s multiple testing correction (p < 0.05, **p** < 0.01, **p** < 0.001). Colour and size of the circles reflect the strength and direction of the correlation, with blue indicating positive and red negative associations. EmDays = days to emergence (days since January 1^th^), FTS = start of flowering (days since January 1^th^), FTE = end of flowering (days since January 1^th^), Ripe = date of picking ripeness (days since January 1^th^), FTdur = duration of flowering (days since January 1^th^), PHFT = plant height at flowering time (cm), PHmin = minimum plant height (cm), PHmax = maximum plant height (cm), PHpod = height of the first pod (cm), HGW = hundred grain weight (g), Surv = survival rate (%), PWSurv = post-winter survival rate (%).


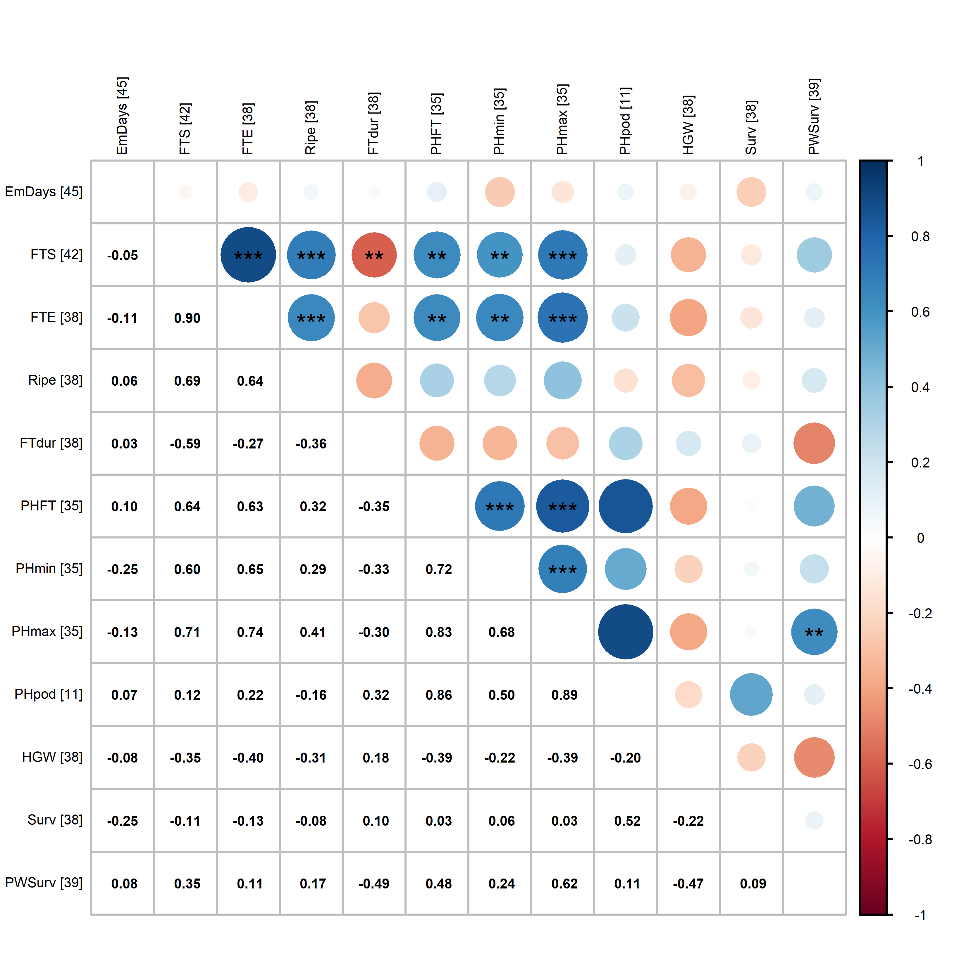

Supplement: Supplementary file 1 — Supplementary file1 (DOCX 2413 kb) [file 122_2025_5032_MOESM1_ESM.docx]
